# Single-Trial Representations of Decision-Related Variables by Decomposed Frontal Corticostriatal Ensemble Activity

**DOI:** 10.1523/ENEURO.0172-24.2024

**Published:** 2024-08-01

**Authors:** Takashi Handa, Tomoki Fukai, Tomoki Kurikawa

**Affiliations:** ^1^Department of Neurobiology, Graduate School of Biomedical and Health Sciences, Hiroshima University, Hiroshima 734-8553, Japan; ^2^Laboratory for Neural Coding and Brain Computing, RIKEN Center for Brain Science, Saitama 351-0198, Japan; ^3^Neural Coding and Brain Computing Unit, Okinawa Institute of Science and Technology, Okinawa 904-0495, Japan; ^4^Department of Complex and Intelligent Systems, Future University of Hakodate, Hokkaido 041-8655, Japan

**Keywords:** frontal corticostriatal ensemble, outcome-based decision-making, single-trial analysis, tensor component analysis

## Abstract

The frontal cortex-striatum circuit plays a pivotal role in adaptive goal-directed behaviors. However, it remains unclear how decision-related signals are mediated through cross-regional transmission between the medial frontal cortex and the striatum by neuronal ensembles in making decision based on outcomes of past action. Here, we analyzed neuronal ensemble activity obtained through simultaneous multiunit recordings in the secondary motor cortex (M2) and dorsal striatum (DS) in rats performing an outcome-based left-or-right choice task. By adopting tensor component analysis (TCA), a single-trial–based unsupervised dimensionality reduction approach, for concatenated ensembles of M2 and DS neurons, we identified distinct three spatiotemporal neural dynamics (TCA components) at the single-trial level specific to task-relevant variables. Choice-position–selective neural dynamics reflected the positions chosen and was correlated with the trial-to-trial fluctuation of behavioral variables. Intriguingly, choice-pattern–selective neural dynamics distinguished whether the incoming choice was a repetition or a switch from the previous choice before a response choice. Other neural dynamics was selective to outcome and increased within-trial activity following response. Our results demonstrate how the concatenated ensembles of M2 and DS process distinct features of decision-related signals at various points in time. Thereby, the M2 and DS collaboratively monitor action outcomes and determine the subsequent choice, whether to repeat or switch, for action selection.

## Significance Statement

We analyzed neuronal ensemble activity simultaneously recorded in the secondary motor cortex (M2) and dorsal striatum (DS) to show how M2-DS circuit mediates decision-relevant signal through cross-regional transmission in decision-making. Decomposed cross-regional neural dynamics exhibited distinct characteristics related to choice position, switch/repetitive choice, and outcome of action at various points in time within trial. These results indicate M2-DS ensemble collaboratively process multiplicate decision-related signals.

## Introduction

Animals can select an appropriate action based on sensory cues and outcomes of past actions to adapt flexibly to changing circumstances (Dolan and Dayan, 2013). The underlying neural circuit is widely believed to involve the frontal cortex-basal ganglia circuit (Ragozzino, 2007; Balleine and O’Doherty, 2010; Hikosaka and Isoda, 2010). However, it remains unclear how population neuronal dynamics interact between the frontal cortex and basal ganglia for the adaptive choice behavior. Synchronous neuronal activity across the frontal cortex and downstream subcortical striatum is correlated with skilled motor learning (Koralek et al., 2013; Lemke et al., 2019) and flexible behaviors based on different rules in a T-maze (Oberto et al., 2022), as well as outcomes of action (Handa et al., 2021). Neural trajectories of the secondary motor cortex (M2), a part of the medial frontal cortex, and dorsal striatum (DS) concomitantly represent choice and outcome information during an outcome-based two-alternative choice task. Precise spike synchrony between M2 and DS neurons becomes more prominent during periods of improved task performance (Handa et al., 2021), suggesting cross-regional coactivation of neuronal population correlated with behavioral variables. However, little is known about how cross-regional neuronal dynamics (i.e., M2-DS ensemble) contribute to decision-making during adaptive outcome-based action selection.

Large-scale neuronal activity can be analyzed by adopting dimensionality reduction methods to profile the neuronal ensemble activity (Cunningham and Yu, 2014). Furthermore, conducting trial-by-trial analyses of ensemble activity provides a means to assess the relationship between alterations in the physiological aspects of neuronal ensemble activity and emerging behavioral variables within a single session (Quian Quiroga and Panzeri, 2009; Musall et al., 2019; Veuthey et al., 2020). Motivational states of an animal regulate behavior and such internal states in the brain can be altered over trials within a single session (Burton et al., 1976; Berridge, 2004; Allen et al., 2019). For example, in the initial trials, a thirsty animal is notably motivated to engage in a behavioral task to attain a drop of water as a reward, whereas its motivated performance may diminish in the later trials. Neural representation in the brain can be altered in accordance with such behavioral changes (Allen et al., 2019). We wonder whether the M2-DS ensemble adaptively processes decision-related information in correlation with behavioral variables during a single behavioral session.

To address these questions, we carried out simultaneous electrophysiological recordings in the M2 and DS while rats performed an outcome-based two-alternative choice task. We analyzed cross-region ensemble spike activity at the single-trial level by applying tensor component analysis (TCA) to the concatenated ensemble activity of M2 and DS neurons. TCA provides a crucial advantage over dimensional reduction approaches, allowing us to quantify trial-to-trial variations in neural activity. Additionally, because TCA is an unsupervised method, unexpected features of ensemble activity can be unveiled (Williams et al., 2018). We observed that choice-position–selective neural dynamics were altered over trials, and such trial-by-trial alterations were correlated with trial-basis behavioral fluctuations. Certain neural dynamics revealed differential states between choice patterns, repetitive choices, and switch choices, even when the incoming motor responses and outcomes were the same. Other neural dynamics could discriminate between the rewarded and unrewarded outcomes following action selection. Choice-pattern–selective within-trial activity differed temporally from outcome-selective within-trial activity. These results suggest that trial-basis fluctuations in M2-DS ensembles could be attributed to behavioral variables and that the M2-DS ensemble was implemented in outcome monitoring and continued decision-making for action selection.

## Materials and Methods

### Animal preparation

All animal procedures were performed in accordance with the Animal Experiment Plan of the Animal Experiment Committee of RIKEN (approval number: H25-2-234[1]). The multiunit recording results during task performance presented in this study were obtained from the reanalysis of previously collected behavioral and electrophysiological data (Handa et al., 2021). Male Long–Evans rats (*N* = 14, 6 weeks, 200–220 g, Japan SLC) were employed. Home cages were situated in a temperature- and humidity-controlled environment with lights maintained on a 12 h light/dark cycle.

### Stereotaxic surgery

All surgical procedures were performed under sterile conditions. Rats were anesthetized with 2% isoflurane, and their body temperature was monitored with a rectal probe and maintained at 37°C on a heating pad during the surgery. A sliding head attachment (Narishige) was implanted in the skull using a dual-curing resin cement (Panavia, Kuraray Noritake Dental) and dental resin (Unifast II, GC). Reference and grounding electrodes (Teflon-coated silver wire, A-M Systems) were positioned over the dura mater above the cerebellum. Following recovery from surgery, rats were deprived of water in their home cages, with water used as a reward for behavioral task execution; however, food was available *ad libitum*. Rats obtained ∼10 ml of water at the task chamber when they engaged in the task performance, whereas they were supplied 10 ml of water at the cage when the behavioral experiment was not performed. To confirm recording sites within regions showing corticostriatal projections from the M2 to DS, a retrograde tracer, Fluoro-Gold (FG; Fluorochrome), was injected into the DS 3 d before the electrophysiological recording experiment. A glass micropipette filled with 2% FG dissolved in 0.1 M cacodylic acid was installed on a micromanipulator angled medially by 27°. The pipette was inserted through a small burr hole drilled in the skull over the left hemisphere (AP: +1.5 mm to the bregma, ML: 1.0 mm to midline, 4.3 mm traveling distance). The pipette tip reached the dorsocentral part of the striatum (AP: +1.5 mm anterior to the bregma, approximately ML: 3.0 mm to midline, ∼3.8 mm ventral to pia mater) based on previous anatomical evidence (Reep et al., 2003). FG was iontophoretically infused using an iontophoresis pump (BAB-501; Kation Scientific). After the completion of training sessions, two cranial windows were created above the DS and M2 of the left hemisphere (AP: +1.0 and +3.0 mm to the bregma, ML: 3.0 and 1.0 mm to midline for DS and M2, respectively), and their dura maters were removed for electrophysiological recordings.

### Behavioral task

**Figure 1.**
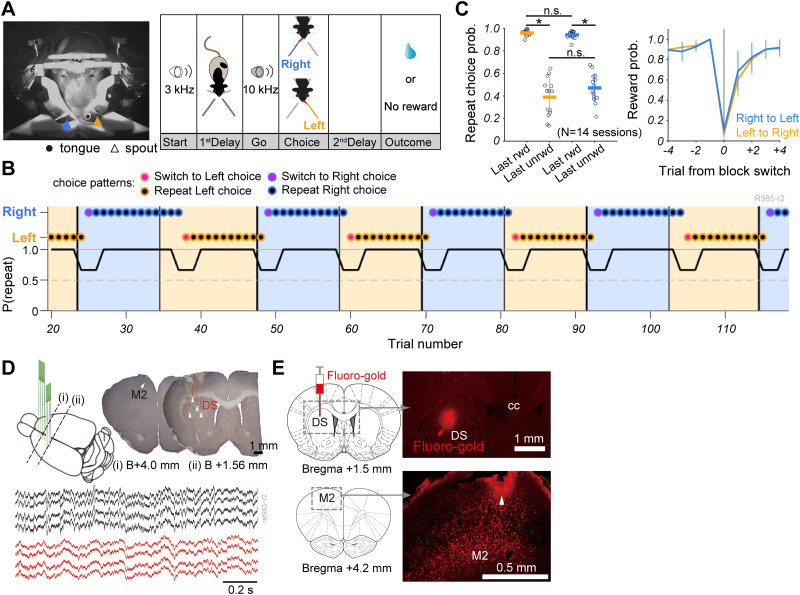
Flexible choice patterns revealing reward-guided repetitive choice and nonreward-guided switch choice. ***A***, Left, Snapshot of a head-fixed rat at the moment of licking choice toward a left spout (orange arrowhead). A black circle and blue arrowhead indicate the position of the tongue and right spout, respectively. Right, Schematic illustration of an outcome-based two-alternative choice task. Each trial began with the presentation of an auditory cue (Start, 3 kHz). Rats awaited another auditory cue (Go, 10 kHz) while abstaining from licking during the initial delay period (1st Delay). Subsequently, they chose either left or right spouts by licking within 5 s. A reward was provided after the second delay period (0.3–0.7 s) if the selected spout position aligned with the current reward position; otherwise, no reward was given, accompanied by a lack of sensory feedback and a 5 s timeout. ***B***, Representative choice pattern revealing repetitive choice behavior postreward acquisition in last trial and switch choice behavior postunrewarded trials. Background colors indicate ongoing reward positions (orange, left spout; blue, right spout). Thick and thin vertical lines denote reversals of reward position: from left to right and from right to left, respectively. Colors of outline and face in symbols represent choice position (left or right) and choice pattern (repetitive or switch), respectively. A line plot displays trial series of the probability of repetitive choice (average probability over three trials). ***C***, Left, Probability of repetitive choice (orange, left choice; blue, right choice) after rewarded (Last rwd) and unrewarded (Last unrwd) outcomes in the last trial. Circles indicate individual sessions (14 sessions, 12 rats). Horizontal lines and error bars represent mean and SD, respectively. Statistical significance was confirmed by one-way ANOVA followed by post hoc Tukey–Kramer test (*p < 0.001). Right, Averaged choice patterns around the reversal of reward position (orange, from left to right; blue, from right to left). Values present mean and SD. ***D***, Top, Schematic illustration of recording sites with two probes in M2 and DS of left hemisphere. Recording sites (white arrowhead) in M2 and DS in the Nissl-stained coronal brain sections. Bottom, Representative local field potentials simultaneously recorded from M2 (black) and DS (red). ***E***, Post hoc confirmation of injection site of retrograde tracer, FG (red arrowhead), in DS and the corticostriatal projection neurons labeled with FG in M2, including the recording site (white arrowhead). cc, corpus callosum. B (or Bregma) indicates the AP coordinate based on the rat brain atlas (Paxinos and Watson, 2009).

Rats underwent training to perform an outcome-based two-choice task using a customized multiple-rat training system (O’Hara & Co.), facilitating parallel learning of the task paradigm for multiple rats simultaneously (Handa et al., 2021). The behavioral task was controlled using a custom-written software in LabVIEW (National Instruments). Individual rats were secured in a body-supporting cylinder, and their heads were rigidly and painlessly fixed using a sliding head holder on a stereotaxic frame (Fig. 1*A*). Spouts were linked to a syringe on a single-syringe pump (AL-1000; World Precision Instruments) using silicon tubing. Water delivery from each spout was regulated by a pinch valve and syringe pump triggered by the TTL signal. The trial initiated with a pure tone presentation (3 kHz, 1 s, 60 dB SPL; Fig. 1*A*, “Start”). Rats were instructed to refrain from licking any spouts from the start cue until the appearance of another auditory cue (10 kHz, 0.2 s, 60 dB SPL; “Go”). If rats licked any spouts during the delay period (“1st Delay”), the trial was promptly aborted. The pseudorandom delay period ranged from 0.7 to 2.3 s. Following the onset of the Go cue, rats could lick either the left or right spouts within a response window (5 s). The first lick was considered as a choice response (“Choice”). If the chosen spout location aligned with the ongoing reward location, 0.1% saccharin water was delivered as a reward after a pseudorandom delay period ranging between 0.3 and 0.7 s (“2nd Delay”). The next trial commenced after an outcome period (4 s, “Outcome”). However, when rats chose a no-reward spout, they received no sensory feedback but had an additional time of 5 s after the outcome period. The subsequent trial began after a timeout. Once the accumulated total number of rewarded trials reached 10 within each block, the reward-associated spout position reversed without any feedback, such as sensory or physical differences in the task. Block reversal occurred after ∼11–12 trials if the rats frequently repeated the rewarded choice. On the other hand, if the rats frequently selected the incorrect spout, the block reversal occurred though >11–12 trials because the block reversal occurred when the rat achieved the criterion (10 rewarded choices within the block). Therefore, rats could not anticipate block reversal without experiencing forthcoming trials. We trained all rats over 21 training sessions. If the performance of rat reached an achievement level (the reward acquisition probability of 75%) within the series of training sessions, we defined the remaining sessions as overtraining sessions. Otherwise, there was no overtraining day. After the training sessions, the rats were subject to recording experiments regardless of the learning achievement.

### Electrophysiological recordings during task performance

Following the training sessions, each animal underwent two daily recording experiments. Multineuron activity was simultaneously recorded from the M2 and DS of the left hemisphere using two 32-channel silicon probes. These probes consisted of four shanks (0.4 mm shank separation), each featuring tetrode-like electrode sites spaced vertically by 0.5 mm (A4×2-tet-7/5mm-500-400-312, NeuroNexus Technologies). Each probe was connected to a custom-made headstage on one of two fine micromanipulators (1760-61, David Kopf Instruments) mounted on a stereotaxic frame (SR-8N, Narishige). The silicon probe was vertically inserted (depth from the pia mater: 1.2 mm) into M2 (at the center of probe: +3.0–3.6 mm to the bregma, 1.0–1.4 mm to midline), with the shanks aligned along the midline (Fig. 1*D*). Another silicon probe, angled posteriorly by 6°, was inserted into DS through a cranial window (at the center of probe: +0.6–1.0 mm to the bregma, 2.7–3.1 mm to midline, 4.0 mm traveling distance), with the shanks aligned along the coronal suture (Fig. 1*D*). Multiunit signals were amplified by the headstages before being fed into main amplifiers (Nihon Kohden) with a bandpass filter (0.5 Hz–10 kHz). All neural data were sampled at 20 kHz using two hard disk recorders (LX-120, TEAC), capturing the time of the task and licking events for each spout (left and right).

### Histology

After the recording sessions, rats were deeply anesthetized with urethane (2–3 g/kg, i.p.) and subsequently perfused intracardially with chilled saline followed by 4% paraformaldehyde (PFA) dissolved in 0.1 M phosphate buffer (PB). The fixed brains were stored in 4% PFA overnight and then placed in a 30% sucrose solution in 0.1 M PB for 2 weeks. Postfixed brains were frozen and coronally sliced into 50-μm-thick serial sections using a microtome cryostat (HM500OM, Microm). The brain sections were stored in 0.1 M PB at 4°C overnight. For fluorescent visualization of FG-labeled neurons, brain sections were incubated with an anti-FG antibody from rabbit (AB153, 1:3,000, Millipore) at 4°C overnight. Subsequently, they underwent incubation with goat anti-rabbit IgG conjugated with Alexa Fluor 594 (A11012, 1:500, Invitrogen) for 2 h. Fluorescence images were acquired using a fluorescence microscope (Olympus, AX70) to confirm the presence of FG-labeled neurons around the silicon probe recording locations in the M2 and near the FG injection site in the DS. To verify the silicon probe track, the slices were counterstained with neutral red Nissl. Recording locations in the M2 and DS as well as AP coordinates were determined in accordance with the rat brain atlas (Paxinos and Watson, 2009).

### Data analysis

All behavioral and neuronal data were analyzed by custom-written MATLAB scripts (The MathWorks).

#### Spike sorting, clustering, and refining

For each tetrode, spikes were isolated from multiunit activity by a custom-made semiautomatic spike-sorting program EToS (12 feature dimensions for four channels; high-pass filter at 300 Hz; time resolution at 20 kHz; spike-detection interval >0.5 ms; Takekawa et al., 2010, 2012). The sorted spike clusters were combined, divided, and discarded manually to refine single-neuron clusters by Klusters (Hazan et al., 2006). To avoid overlapping of detection of same units recorded between distinct tetrodes, we checked cross-correlations of spike times among isolated units across all of tetrodes. If there was a high correlation peak only at zero time between a pair of units, one of the units was excluded from further analyses because those spikes which originated from the same neuron were presumably recorded through different tetrodes.

#### Dataset

We reanalyzed behavioral and multineuron spike datasets previously recorded, sorting them in 14 recording sessions with 12 rats using a new analytical approach (Handa et al., 2021). Dataset for this study is summarized in Table 1, where the experimental ID, number of overtraining sessions, reward acquisition probability (the fraction of correct choices) in the recording session, and number of M2 and DS units are listed.

**Table 1.** Dataset

Session ID	Number of overtraining sessions	Reward acquisition probability	Number of M2 neurons	Number of DS neurons
R982-r1	7	*p *= 0.828	*N* = 45	*N* = 43
R983-r1	8	*p *= 0.837	*N* = 35	*N* = 55
R983-r2	9	*p *= 0.834	*N* = 58	*N* = 48
R985-r1	10	*p *= 0.853	*N* = 30	*N* = 60
R985-r2	11	*p *= 0.867	*N* = 66	*N* = 26
R986-r1	4	*p *= 0.813	*N* = 16	*N* = 20
R991-r1	7	*p *= 0.806	*N* = 46	*N* = 31
R997-r1	0	*p *= 0.740	*N* = 24	*N* = 34
R1000-r1	1	*p *= 0.784	*N* = 12	*N* = 32
R1002-r1	3	*p *= 0.744	*N* = 31	*N* = 11
R1004-r1	1	*p *= 0.792	*N* = 20	*N* = 51
R1005-r1	4	*p *= 0.794	*N* = 65	*N* = 64
R1009-r1	3	*p *= 0.774	*N* = 35	*N* = 33
R1012-r1	6	*p *= 0.755	*N* = 40	*N* = 26

#### Tensor component analysis

**Figure 2.**
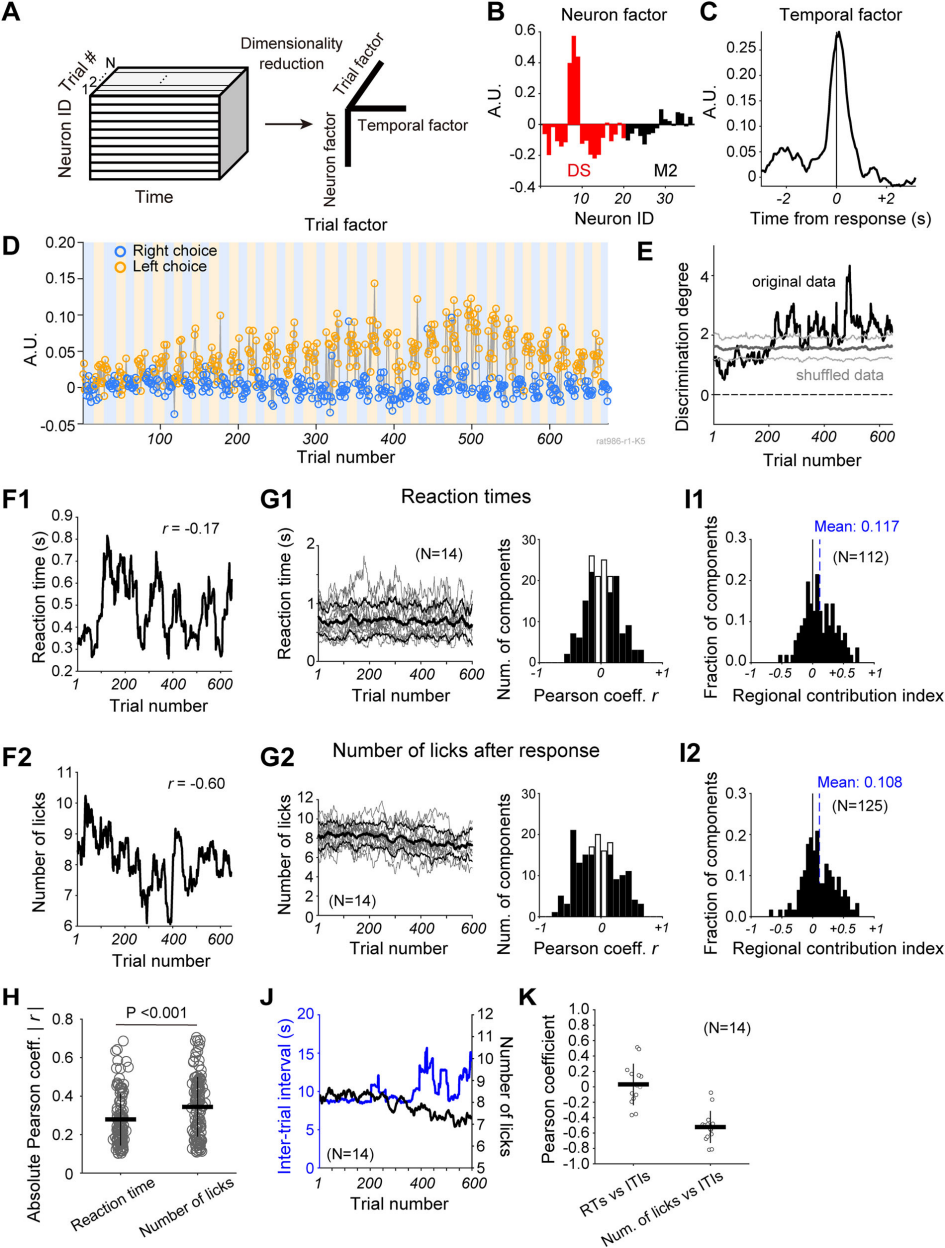
Trial-to-trial changes in choice-position–selective TCA component of M2-DS ensemble activity are related to behavioral variables. ***A***, A schematic illustration of TCA. ***B–D***, Example of a TCA component. ***B***, Neuron factor, including M2 (black) and DS (red) neurons. ***C***, Temporal factor (within trial activity). ***D***, Trial factor of the TCA component. Symbol colors denote the choice position at each trial (left, orange; right, blue). Background colors represent the ongoing reward position (orange, left spout; blue, right spout). ***E***, Trial series of discrimination degree of the trial factor shown in ***D*** using the original trial order (black) and shuffled trial order (gray, mean and SD of discrimination degree acquired by repeating shuffling trial order). ***F1***, Trial series of reaction times (RTs) and (***F2***) trial series of the number of licks after the choice response in the same session as shown in ***B–D***. “*r*” denotes Pearson's correlation coefficient. ***G1***, Left, Trial-series of RTs over 14 sessions. Gray and black lines represent individual and averaged values. Error range indicates SD. Right, The distribution of correlation coefficients between discrimination degree and RTs. ***G2***, Left, Trial series of the number of licks after the choice response over 14 sessions. Right, The distribution of correlation coefficients between discrimination degree and the number of licks. Open and filled bars indicate the number of nonsignificant and significant correlation coefficients, respectively (Pearson's correlation; *p *< 0.01). ***H***, Comparison of absolute correlation coefficients showing statistical significance (as shown in ***G1*** and ***G2***) between two behavioral variables. Statistical significance was assessed using a two-sample *t* test. Horizontal and vertical lines represent mean and SD, respectively. ***I***, The distribution of regional contribution indices for TCA components which correlated with RTs (***I1***) and with the number of licks (***I2***). The vertical dashed line indicates mean value. Statistical significance was assessed using *t* test. ***J***, The mean trial series of ITIs (blue) with the mean trial series of the number of licks (black) as shown in ***G2***. ***K***, Correlation coefficients between behavioral variables for all 14 recording sessions. Horizontal and vertical lines represent mean and SD, respectively.

To generate an original neural data tensor *X* for each recording session, we computed trial-based perievent time histograms aligned at the choice response, using a 200 ms sliding window with a 50 ms step for individual M2 and DS neurons. To obtain substantial firing activity in population of neurons, we chose the 200 ms bin for the sliding window because the signal-to-noise ratio in characteristics of TCA components using 200 ms bin was better than that analyzed using 50 ms bin for the sample size of our data. Subsequently, we obtained a third-order tensor (neuron, time, and trial) for the M2-DS ensemble through TCA (Fig. 2*A*). To investigate the single-trial dynamics of the M2-DS ensemble activity, we applied TCA to tensor *X* using a MATLAB-based toolbox (Tensor Toolbox for MATLAB, version 3.2.1, https://www.tensortoolbox.org/; Bader and Kolda, 2008). In this analysis, the M2-DS ensemble activity was decomposed into a third-order tensor $X_{ntk}=\sum_{r=1}^{R}\;w_{n}^{r}b_{t}^{r}a_{k}^{r}$ by the summation of one-rank components (Fig. 2*A*). Each component consists of three vectors: $w_{n}^{r}$ represents the *n*-th element of a “neuron factor” vector, reflecting a prototypical firing rate pattern across neurons; $b_{t}^{r}$ is the *t*-th element of a “temporal factor” vector, representing a temporal basis function across time; and $a_{k}^{r}$ is the *k*-th element of a “trial factor” vector, signifying a trial-specific bias for spatiotemporal activity in a trial. We set the number of TCA components *R* to 15 for canonical polyadic decomposition based on a previous study, which suggested that 15 components were sufficient to profile the population of neurons encoding task variables in a behavioral experiment (Williams et al., 2018). For the analysis of ensemble coding in M2 and DS alone, we applied TCA to the M2 and DS ensembles alone, respectively.

#### Choice-position selectivity while trials progress

We assessed significant differences in neural dynamics between left and right choice trials in a given session by applying a two-sample *t* test (*p *< 0.05) to trial factors for each TCA component. If the differences were statistically significant, we referred to the TCA component as “choice-position” selective TCA component. We computed the discrimination degree *d*’ as a choice-position selectivity index in a window of 30 trials by sliding the window with one trial step. Instantaneous *d*’ was computed as follows:

$$D=\sqrt{{\left(a_{L}-a_{R}\right)}^{2}\;/(\sigma_{L}^{2}+\sigma_{R}^{2})},$$

where *a_L_* and *a_R_* indicate the means of the trial factors of the left and right trials within 30 trials, respectively. *σ_L_* and *σ_R_* denote the standard deviations (SD) of the trial factors of the left and right trials within 30 trials, respectively.

$$\mathrm{If}\;a_{L}-a_{R}>0,d^{\prime }=+D,$$

$$\mathrm{If}\;a_{L}-a_{R}<0,d^{\prime }=-D.$$

To assess whether the trial-wise discrimination degree (original *d*’) was altered across trials, we used a permutation test by comparing it with the control data. As a control discrimination degree, we randomly shuffled the trial order and computed the trial-wise of discrimination degree (surrogated *d*’) by means of the shuffled trial factor. Subsequently, the original *d*’ was compared with the surrogated *d*’ by paired *t* test (*p *< 0.05). This procedure was repeated 100 times. If a statistically significant difference was observed in >95% of 100 repetitions, we determined that the TCA component altered the choice-position selectivity across trials.

#### Correlation of choice-position selectivity with behavioral variables

To investigate whether the change in choice-position selectivity of the TCA component was correlated with the change in behavioral variables, we calculated two behavioral variables: reaction times (RTs) and the number of licks after the choice response. In each trial, RT was calculated as the duration between Go cue onset and the time at which the first lick occurred after Go cue onset and the number of licks that emerged within 2 s after the choice response. We computed the average RTs and number of licks by sliding the analysis window of 30 trials in one trial step. We subsequently computed Pearson's correlation between the trial series of choice-position selectivity *d*’ and each behavioral variable. If the *p* value was <0.01, we defined the TCA component as significantly correlated with the behavioral variable.

#### Trial series of intertrial intervals

We measured a duration between the lick choice at trial *t* and the onset of Start cue onset at the trial *t* + 1, in which the rat engaged in task performance (lick choice) after Go cue onset. We defined this duration as an intertrial interval (ITI). If the rat did not make the lick choice at the trial *t* + 1, we repeated the same trial until the animal made response and thus the ITI got longer. The ITIs were averaged by sliding the analysis window of 30 trials in one trial step as treated for trial series of RTs and the number of licks above.

#### Quantification of choice-pattern selectivity in M2-DS combined ensemble activity

To quantify the extent to which the neural dynamics in switch choice trials differed from those in repetitive-choice trials, we computed the standard score (*Z*-score) of trial factors based on their mean and SD within individual blocks. In the case of the left block, during which the left spout was associated with reward delivery, we calculated the *Z*-score of trial factors in individual left blocks, ranging from the trial where the rat switched its choice from the preceding unrewarded choice (switching from right unrewarded choice to left rewarded choice) to the last trial before the reversal of reward position. The *Z*-scores in the switch choice trial (Switch) and in the three continued repetitive-choice trials (Rep-1, Rep-2, and Rep-3) were computed as follows:

$$Z_{i}=(a_{i}-m)/\sigma ,$$

where *a_i_*, *m*, and *σ* indicate the trial factor (*i *= Switch, Rep-1, Rep-2, or Rep-3 choice trial) in a block and the mean and SD of the trial factors within the block, respectively. To derive *m* and *σ* in individual blocks (e.g., the left reward block), we used trials that included the same choice (left choice) and outcome (rewarded) conditions, excluding other choice conditions (right choice and unrewarded). In a given block, if any unrewarded choice trials intermingled before the four continued rewarded choices were achieved, the block was removed from this analysis. We assessed the statistical significance of differences in *Z*-scores among the four conditions (Switch, Rep-1, Rep-2, and Rep-3) by one-way ANOVA, followed by post hoc multiple-comparisons Dunnett's test (*p *< 0.05). If all of three pairs (Switch vs Rep-1, Switch vs Rep-2, and Switch vs Rep-3) exhibited significance, we categorized the TCA component as a “choice-pattern”–selective TCA component. The same computation was performed separately for the right reward block.

#### Quantification of outcome selectivity in M2-DS combined ensemble activity

To quantify the level of trial factor differences between rewarded and unrewarded choice trials in M2-DS combined ensemble activity, *Z*-scores of trial factors were computed, similar to the aforementioned analysis of choice-pattern selectivity. For the left block, trial factors were collected in a sequence of trials beginning from a trial where the rat correctly switched its choice from the preceding unrewarded choice (right to left) up to one trial before the initial switch choice trial (left to right) in the next block. This chunk of trials encompassed both repetitive-choice but unrewarded trials, occurring due to the reversal of the reward position. *Z*-scores were computed for the initial three repetitive choice and reward trials (Rep-1 & rwd, Rep-2 & rwd, and Rep-3 & rwd), along with those of the repetitive choices and unrewarded trials (Rep & unrwd), as described earlier. If any unrewarded choice trials were interspersed before achieving the four consecutive rewarded choices, the block was excluded from the analysis. Assuming that Rep-1 and rwd, Rep-2 and rwd, and Rep-3 & rwd and Rep and unrwd represented the same choice pattern (repetitive choice) but different outcome conditions (Rep-1 & rwd, Rep-2 & rwd, and Rep-3 & rwd: rewarded; Rep & unrwd: unrewarded). Statistical significance was determined through one-way ANOVA among the four choices, followed by post hoc multiple-comparisons Dunnett test (*p *< 0.05). If all three pairs (Rep & unrwd vs Rep-1 & rwd, Rep & unrwd vs Rep-2 & rwd, and Rep & unrwd vs Rep-3 & rwd) exhibited significance, the TCA component was categorized as “outcome”-selective TCA component. A parallel computation was conducted for right choice trials separately.

#### Quantification of choice-pattern and outcome selectivity in M2- and DS-alone ensemble activities

TCA was applied separately to the M2- and DS-alone ensemble activities using the same dataset as the M2-DS ensemble activity. *Z*-scores were computed for the analyses of choice-pattern and outcome selectivity, following the previously described methods. To compare the TCA components based on the M2- and DS-alone ensemble with those based on M2-DS ensemble, we randomly resampled the equivalent number of neurons in the M2-DS ensemble relative to the number of neurons in M2- and DS-alone ensemble, respectively. TCA was applied to the M2-DS ensemble to get TCA component. The number of TCA components and their *Z*-scores were compared with the TCA data based on M2-/DS-alone ensemble. When we compared *Z*-scores between M2-DS ensemble and M2-/DS-alone ensemble, we used two-sample *t* test to obtain *t*-statistics. We repeated this procedure 10 times. If *t* statistic was positively larger than 0, the *Z*-score of the M2-DS ensemble was larger than that of the M2-/DS-alone ensemble and vice versa.

#### Quantification of regional contribution in M2-DS combined ensemble activity

To quantify the contribution of DS and M2 to the TCA component based on M2-DS combined activity, we computed the regional contribution index using neuron factor. The values of each neuron factor were separated into values for M2 and DS cells, and their root mean square (RMS) was computed for each region. The regional contribution index *C* was computed as follows.

$$C=(S_{\mathrm{DS}}-S_{M2})\;/(S_{\mathrm{DS}}+S_{M2}),$$

where *S*_DS_ and *S*_M2_ indicate the RMS of neuron factor for DS cells and RMS of neuron factor for M2 cells.

## Results

### Rats exhibit retrospective outcome-based choices

Head-restrained rats engaged in an outcome-based two-choice task, selecting between two spouts to make their choice (Fig. 1*A*). A reward was received when the chosen spout matched the ongoing reward-spout position. The action–outcome association was systematically reversed without sensory feedback after accumulating 10 rewarded trials in each block. We analyzed the behavioral data from 14 recording sessions with 12 rats. Across all sessions, the average number of trials per session was 589 ± 124 (mean ± SD). Rats discerned the reversal of choice-reward contingency within subsequent trials, responding to the experience of no-reward events, time-out, and reward acquisition (Fig. 1*B*). The averaged trial number per block was 12.5 ± 0.650 (mean ± SD). In both the left and right choice trials, rats repeatedly selected the same spout as that in the preceding rewarded trial but switched their choice following one to several unrewarded trials (Fig. 1*B*,*C*; one-way ANOVA: *p *< 10^−21^, post hoc Tukey–Kramer test: last rewarded trials vs last unrewarded trials, left choice, *p *< 0.001, right choice, *p *< 0.001). No statistically significant differences were observed in the repetitive choice probabilities between the left and right choice trials regardless of the outcome conditions in the preceding trial (post hoc Tukey–Kramer test: last rewarded trials, left choice vs right choice, *p *= 0.973; last unrewarded trials, left choice vs right choice, *p *= 0.201). The rats demonstrated an inability to predict the reversal of the reward block, choosing nearly no reward-associated spouts in some trials postreversal. In response to the block reversal, rats then retrospectively switched to another choice (Fig. 1*C*, right). In essence, the choice pattern of the rats closely mirrored the win-stay and lose-shift strategy, an optimal approach for maximizing reward acquisition in the current task, considering that the rats did not anticipate the block reversal.

### Simultaneous recording of ensemble neural activity from M2 and DS

To investigate whether frontal corticostriatal ensembles are encoded at the single-trial level, we conducted simultaneous recordings of multineuron activity in both the M2 and DS of the left hemisphere using two multielectrode probes (Fig. 1*D*). Across 14 recording sessions, a considerable number of units were recorded in both M2 (mean ± SD, 37.3 ± 16.5 units) and DS (38.8 ± 15.5 units). The rodent M2 (or the rostral agranular medial cortex) is one of the primary cortical areas projecting to the dorsocentral region of the striatum (Cheatwood et al., 2003; Reep et al., 2003; Hintiryan et al., 2016). To confirm the presence of such corticostriatal projections from the recording site in M2 to that in the DS, we iontophoretically infused a retrograde tracer FG into the central part of the DS before recording sessions (Materials and Methods). FG-labeled corticostriatal neurons were primarily observed in layers 3 and 5 of the M2 (Fig. 1*E*). The probe track for M2 and DS recordings aligned with the FG-labeled region in the M2 or near the injection site in the DS (Fig. 1*D*,*E*), confirming the recording of the multineuron activity from the directly connected subregions of the M2 and DS.

### Trial-by-trial changes in choice-position–selective activity of M2-DS ensembles are correlated with behavioral variables

To investigate the dynamic changes in the characteristics of M2-DS ensembles across trials, we utilized TCA (Williams et al., 2018) for both dimensionality reduction of high-dimensional neuronal activity and quantification of trial-to-trial fluctuations in ensemble activity (Materials and Methods). Here, we refer to the collective firing activity of multiple M2 and DS neurons as the M2-DS ensemble. TCA decomposes the M2-DS ensemble activity into a third order tensor (Fig. 2*A*). Each component comprises three vectors, including a “neuron factor” vector (Fig. 2*B*), a “temporal factor” vector (Fig. 2*C*), and a “trial factor” vector (Fig. 2*D*).

We hypothesized that the TCA components of the M2-DS ensemble would exhibit choice-position selectivity that changes across trials, consistent with our previous findings of choice-position selectivity in single-unit and population activity in both the M2 and DS during this task (Handa et al., 2021). Indeed, the trial factor of this TCA component revealed significant differences between the left and right choices (two-sample *t* test; *p *< 10^−126^). Additionally, we observed that the difference in trial factor between choice positions was small in the initial trials but gradually increased in the middle and later parts of this session (Fig. 2*D*). To quantify the gradual changes in trial factors across trials, we calculated the degree of discrimination across trials as choice-position selectivity (Materials and Methods). The discrimination degree increased across trials and significantly differed from the discrimination degree derived by shuffling trial order (permutation test; *p *< 0.01; Materials and Methods; Fig. 2*E*).

We examined whether the gradual changes in the discrimination degree were associated with alterations in behavioral variables across trials. Specifically, we investigated the relationship between the discrimination degree and two behavioral variables: reaction time (RTs) and the number of licks after the choice response. The trial series of the number of licks exhibited a higher correlation with the degree of discrimination of the TCA component (Pearson's correlation: *r *= −0.60; *p *< 0.0001) than with the trial series of RTs (*r *= −0.17; *p *< 0.0001; Fig. 2*F*). Although RTs (Fig. 2*G1*) and the number of licks (Fig. 2*G2*) varied across the 14 sessions, an average reduction in the number of licks was observed across trials. The slope of linear regression model, which was fitted to the trial series of the number of licks for each session, was negative (slope, mean ± SD = −0.00192 ± 0.00101) and significantly different from 0 (*t* test; *p *= 8.20 × 10^−6^). Among the 166 choice-position–selective TCA components, 112 (67.4%) and 125 (75.3%) were significantly correlated with RTs and the number of licks, respectively (Pearson's correlation; *p *< 0.01; Fig. 2*G1*,*G2*, right). Among these significantly correlated TCA components, 87 revealed a significant correlation with both RTs and the number of licks. The magnitudes of the significant correlation coefficients were larger in the correlation with the number of licks than in the correlation with RTs (two-sample *t* test; *p *< 0.001; Fig. 2*H*). These behavior-correlated TCA components were observed in all recording sessions. Their correlation coefficients were not significantly correlated with the number of overtraining sessions on this task (Pearson's correlation test: correlation with RTs: *r *= −0.160, *p *= 0.583; correlation with the number of licks: *r *= 0.024, *p *= 0.933), suggesting that the properties of trial factors in the choice-position–selective TCA were not affected by the overtraining for this task.

To quantify how much each brain region contributed to the choice-position–selective TCA components which correlated to RTs or the number of licks, we computed the regional contribution index using neuron factor (Materials and Methods). The regional contribution indices broadly ranged (Fig. 2*I1*,*I2*, respectively). On average, the contribution index was significantly shifted to positive values, suggesting the DS was more of a contributor than the M2 (*t* test: TCA components correlated to RTs: *p *= 2.81 × 10^−6^, TCA components correlated to the number of licks: *p *= 1.03 × 10^−5^).

In contrast to the reduction tendency of number of licks at the late trials in the sessions, we found that ITIs, which were intervals between consecutive trials where animals were engaged in lick response post Go cue, got longer at the late trials in the sessions (Fig. 2*J*; slope, mean ± SD = 0.00659 ± 0.00806, *t* test: *p *= 0.00912). If the rat did not respond to Go cue within the response window, the same trial was repeated until the rat made choice, resulting in longer ITIs. This increasing tendency of ITIs at the late trials indicates the reduction of motivation to engage in task performance. Thus, the ITIs could be a kind of behavioral variable to quantify how constantly animals were motivated to engage in choice action to obtain a reward. The number of licks were negatively correlated with the ITIs (Pearson's correlation coefficient: mean ± SD = −0.521 ± 0.20751, *t* test: *p *= 3.62 × 10^−7^; Fig. 2*J*,*K*), but RTs were not (mean ± SD = 0.0307 ± 0.262, *t* test: *p *= 0.676; Fig. 2*K*). Therefore, the behavioral results suggest that this reduction in the number of licks over the trials may reflect changes in the motivational state of the rats to engage in task performance.

These results indicate that TCA unveils the dynamics of choice-position selectivity in M2-DS ensembles across trials, which may be linked to changes in behavioral variables such as motor preparation and/or motivational state.

### TCA unveils activity patterns of M2-DS ensembles distinguishing between repetitive and switch choices

**Figure 3.**
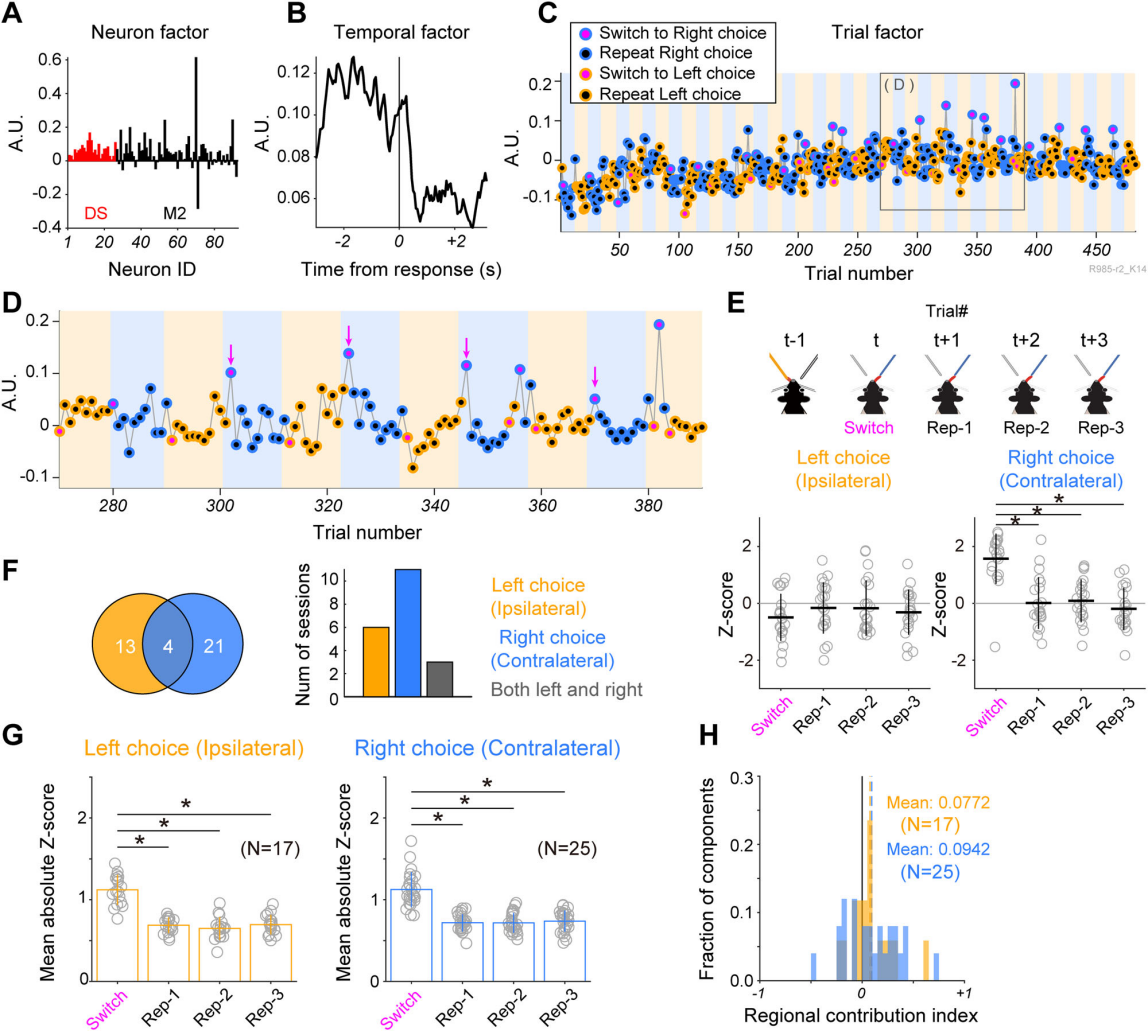
Trial order activity pattern of M2-DS ensembles related to repetitive and switch choices. ***A***, Neuron factor, (***B***) temporal factor, and (***C***) trial factor of a representative TCA component revealing large variance in switch choice trials. Edge and face colors represent choice positions (orange, left; blue, right) and choice patterns (black, repetitive choice; magenta, switch choice), respectively. Background colors denote ongoing reward positions (orange, left spout; blue, right spout). A gray box presents an area enlarged in ***D***. ***D***, Representatives of switch choice trials (magenta arrows) exhibit significantly larger variance when the ongoing reward position is at the right spout. ***E***, Top, A schematic illustration of choice patterns utilized for the statistical estimation of differences in trial factors among repetitive and switch choices. This trial sequence illustrates a switch from left to right choices after the reversal of reward position (from the left spout to right the spout). At trial *t*, the animal shifts its choice to the right spout, transitioning from the left choice selected in the preceding trial (*t *− 1). Subsequent repetitive choices (*t* + 1, *t* + 2, and *t* + 3) are employed to compare differences among switch (Switch) and repetitive (Rep-1, Rep-2, and Rep-3) choice patterns. Bottom, *Z*-scores of trial factors in Switch, Rep-1, Rep-2, and Rep-3 choice patterns within the same dataset as depicted in ***C*** and ***D***, corresponding to the left (ipsilateral) and right (contralateral) choices of the rat. The statistical significance of differences is assessed through one-way ANOVA followed by post hoc Dunnett test (*p < 0.05). Horizontal and vertical lines represent mean and SD, respectively. ***F***, The Venn diagram illustrates the number of TCA components with significantly different *Z*-scores of trial factors between repetitive choice and switch choice trials in left choice (orange), right choice (blue), and both (merge) conditions. The bar graph indicates the number of sessions featuring TCA components with significantly different *Z*-scores of trial factors between repetitive and switch choice trials. ***G***, Population data of TCA components reveal a significant difference in *Z*-scores between repetitive and switch choice trials. Individual dots indicate the mean of absolute *Z*-score per TCA component. Bar graphs show the mean and SD. Statistical significance of the difference is assessed through one-way ANOVA followed by post hoc Dunnett test (*p < 0.05). ***H***, The distribution of regional contribution indices for TCA components which were significantly different between switch and repetitive choices in left choice block (orange) and right choice block (blue). The vertical dashed line indicates mean value. Statistical significance was assessed using *t* test.

In this study, TCA not only confirmed the anticipated choice-position–selective activity type of M2-DS ensembles, as revealed in our previous analysis (Handa et al., 2021), but also unveiled an unexpected trial factor pattern that differentially altered depending on the choice pattern—switch choice versus repetitive choice (Fig. 3*A–C*). A representative TCA component exhibited an increase and decrease in within-trial activity (temporal factor) before and after the response, respectively (Fig. 3*B*). The trial factor displayed distinct patterns between repetitive and switch choices in right choice trials but not in left choice trials (Fig. 3*C*). In right choice trials, the trial factors in switch trials significantly deviated from those in the repetitive trials (Fig. 3*C*,*D*). In contrast, in left choice trials, the trial factor fluctuated similarly in both switch and repetitive choices. To quantify the deviation within a single block of trials, we calculated the *Z*-scores of trial factors within each block to compare switch choice, first, second, and third repetitive choices, where the movement direction and outcome condition (rewarded) were the same (Fig. 3*E*, top; Materials and Methods). The *Z*-scores in switch choice trials significantly differed from those in all other repetitive choice trials in the right (contralateral) choice condition (one-way ANOVA: *p *< 10^−13^, post hoc Dunnett test, Switch vs Rep-1: *p *< 0.0001, Switch vs Rep-2: *p *< 0.0001, Switch vs Rep-3: *p *< 0.0001), whereas the *Z*-score was not significantly different in the left (ipsilateral) choice condition (one-way ANOVA: *p *= 0.102; Fig. 3*E*). If the difference in *Z*-scores between switch and repetitive choices reflected difference in previous choice positions (or previous outcomes), the difference in *Z*-score should be observed in both left and right choice conditions. Therefore, this lateralized difference between repetitive and switch choices could not be attributed to the differences in the previous choices or outcomes between the choice patterns, suggesting that this difference may reflect rather a lateralized cognitive function for switch or repetitive choices. Choice-pattern–selective TCA components were more frequently observed in right (contralateral) choice trials (*N* = 21) than in left (ipsilateral) choice trials (*N* = 13). Four TCA components displayed choice-pattern–selective activity in both left and right choice trials. The right choice-preferred TCA component (11 sessions) was detected in more recording sessions than the left choice-preferred TCA component (six sessions; Fig. 3*F*). The TCA component revealing choice-pattern selectivity in both left and right choices was observed in three sessions. The population of *Z*-scores for the choice-pattern–selective TCA trial factor was significantly higher in switch choice trials than in continued repetitive choice trials (Fig. 3*G*). The regional contribution indices broadly ranged (Fig. 3*H*). On average, the contribution index was not different from 0 (*t* test: *p *= 0.135 for left choice block, *p *= 0.0911 for right choice block), suggesting the M2 and DS equally contributed to the choice-pattern–selective TCA components.

This result suggests that the M2-DS ensembles differentially encode incoming choice information between switch choice (left to right) and repetitive choice (right to right) at the single-trial level.

### TCA component exhibits a differential magnitude depending on rewarded and unrewarded choices

**Figure 4.**
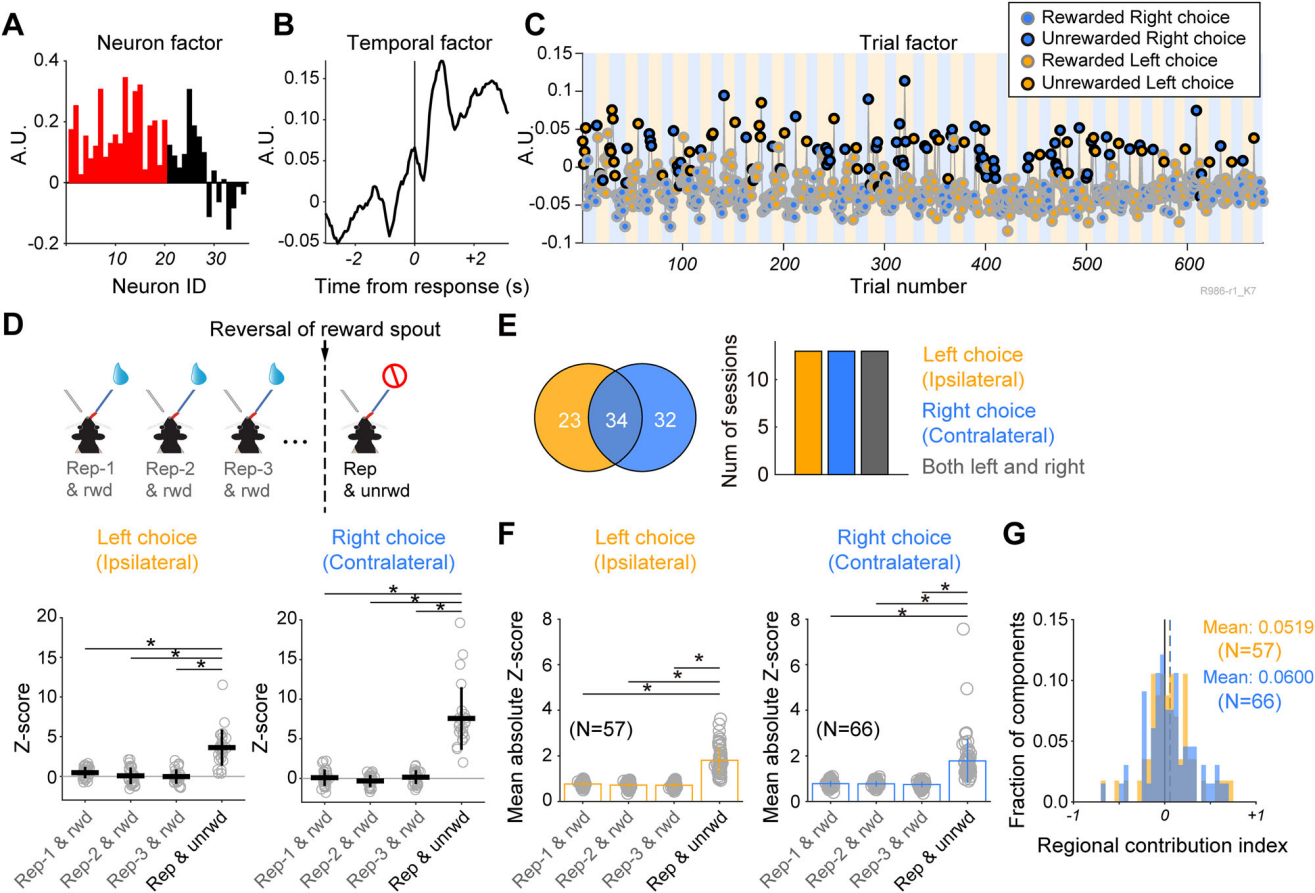
Trial factor of a TCA component distinguishing between rewarded and unrewarded choice trials. ***A***, Neuron factor, (***B***) temporal factor, and (***C***) the trial factor of the TCA component, revealing substantial variance during unrewarded choice trials. In the trial factor, different colors represent choice patterns, with edge and face colors denoting outcomes (rewarded, gray; unrewarded, black) and choice positions (orange, left; blue, right), respectively. ***D***, Top, A schematic illustration of outcome conditions used for statistical estimation of differences in trial factors of repetitive choice trial between rewarded and unrewarded outcomes. A vertical dashed line indicates the reversal of reward position from the right spout to the left spout. The trial sequence illustrates a series of repetitive choice trials after a switch choice trial with a rewarded outcome (Rep-1 & rwd, Rep-2 & rwd, and Rep-3 & rwd), as well as repetitive choice trials without reward outcomes after the reversal of the reward position (Rep & unrwd). Bottom, *Z*-scores of trial factors computed in Rep-1 & rwd, Rep-2 & rwd, and Rep-3 & rwd and Rep & unrwd are shown in the session depicted in panel ***C*** when the animal made left and right choices. The statistical significance of the difference is assessed through one-way ANOVA followed by post hoc Dunnett test (*p < 0.05). ***E***, The Venn diagram displays the number of components revealing significantly different trial factors between rewarded and unrewarded repetitive choice trials at left choice (orange), right choice (blue), and both (merge). The bar graph indicates the number of sessions where components showed significant differences between rewarded and unrewarded repetitive choice trials. ***F***, Population data of TCA components revealing a significant difference in *Z*-scores between rewarded and unrewarded repetitive choice trials. Individual dots represent the mean of absolute *Z*-score per TCA component, with bar graphs depicting the mean and SD. Statistical significance was assessed through one-way ANOVA followed by post hoc Dunnett test (*p < 0.05). ***G***, The distribution of regional contribution indices for TCA components which were significantly different between rewarded and unrewarded repetitive choices in left choice block (orange) and right choice block (blue). The vertical dashed line indicates mean value. Statistical significance was assessed using *t* test.

In our previous study, both M2 and DS ensembles displayed outcome-related activity (Handa et al., 2021). As expected, the TCA of the M2-DS ensemble revealed a change in trial factors depending on the outcome (rewarded and unrewarded events) at the single-trial level (Fig. 4*A–C*). A representative TCA component showcased an increase in within-trial activity (temporal factor) following the response (Fig. 4*B*). The trial factor highly deviated in unrewarded choice trials without a bias of laterality (Fig. 4*C,D*; left choice: one-way ANOVA: *p *< 10^−18^, post hoc Dunnett test, Rep-1 & rwd vs Rep & unrwd: *p *< 0.0001, Rep-2 & rwd vs Rep & unrwd: *p *< 0.0001, Rep-3 & rwd vs Rep & unrwd: *p *< 0.0001; right choice: one-way ANOVA: *p *< 10^−27^, post hoc Dunnett test, Rep-1 & rwd vs Rep & unrwd: *p *< 0.0001, Rep-2 & rwd vs Rep & unrwd: *p *< 0.0001, Rep-3 & rwd vs Rep & unrwd: *p *< 0.0001). Outcome-selective TCA components were frequently observed in both left and right choice trials (Fig. 4*E*), in contrast to the choice-pattern–selective TCA component discussed earlier. The population of *Z*-scores for the outcome-selective TCA trial factor was significantly higher in the repetitive but unrewarded choice trials than in the repetitive and rewarded choice trials (Fig. 4*F*). The regional contribution indices broadly ranged in both left and right choice blocks (Fig. 4*G*). The mean contribution index was not different from 0 (*t* test: *p *= 0.138 for left choice block, *p *= 0.0700 for right choice block), suggesting the M2 and DS equally contributed to the outcome-selective TCA components.

This finding suggests that the M2-DS ensembles differentially encoded outcome information in a given trial.

### Differential within-trial activity in choice-pattern–selective and outcome-selective TCA components

**Figure 5.**
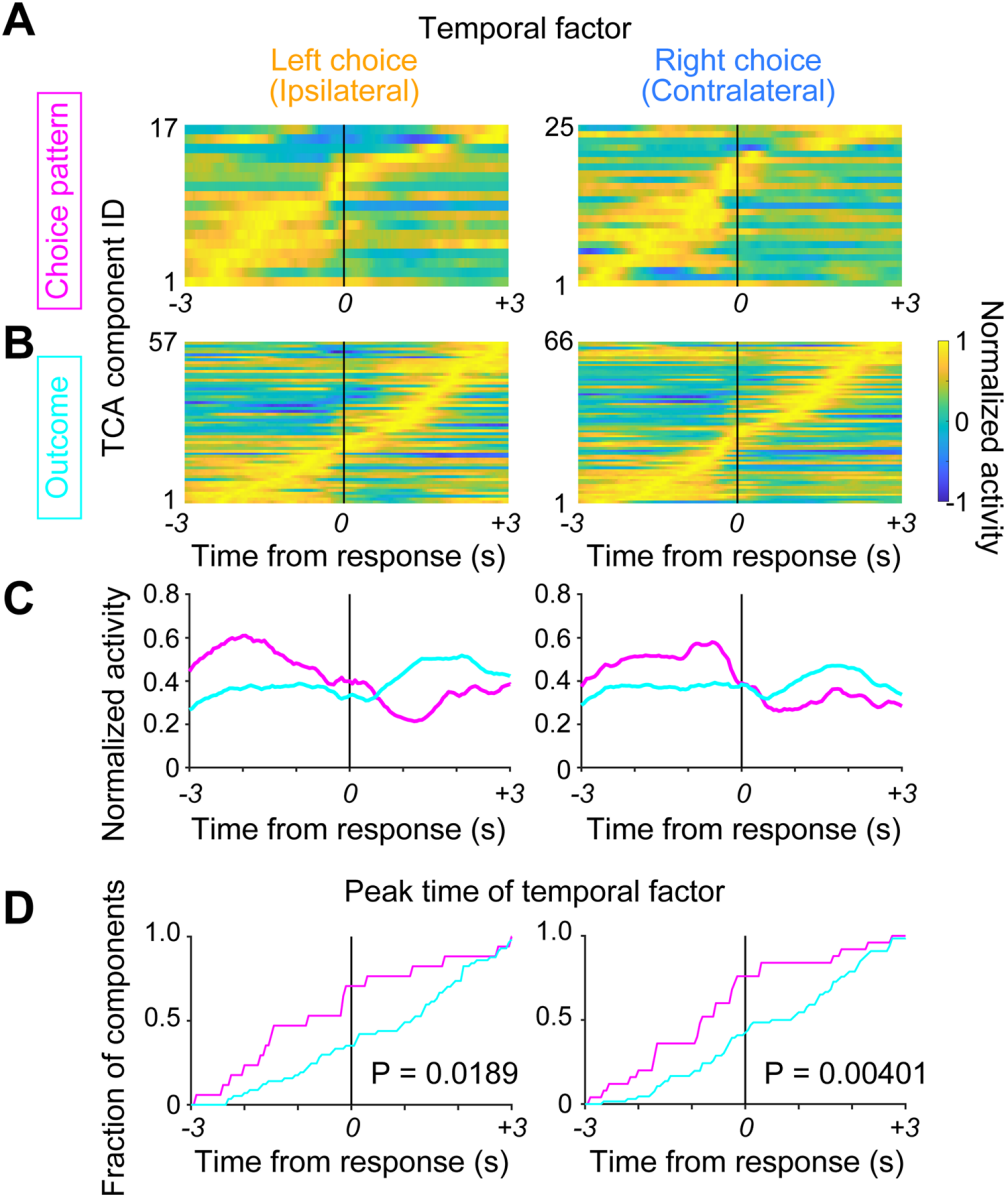
Differential within-trial activity of M2-DS ensembles between choice-pattern–selective and outcome-selective TCA components. ***A***, Collective normalized temporal factors (within-trial activity) of TCA components with a significant difference in *Z*-scores of trial factor between repetitive and switch choice trials at left (ipsilateral) and right (contralateral) choice trials. ***B***, Collective normalized temporal factors of TCA components exhibit a significant difference in *Z*-scores of trial factors between rewarded and unrewarded repetitive choice trials at left (ipsilateral) and right (contralateral) choice trials. ***C***, Averaged normalized temporal factors related to choice patterns (magenta) and outcomes (cyan). ***D***, Cumulative summation curve of peak time of temporal factors of choice-pattern–selective TCA components (magenta) and outcome-selective TCA components (cyan). Statistical significance was assessed using the Mann–Whitney *U* test.

The functionally distinct TCA components likely reflect characteristics revealing different roles of the M2-DS ensemble in adaptive outcome-based decision-making. To address this question, we examined the differences in within-trial activity (temporal factor) between the choice-pattern–selective and outcome-selective TCA components. Most choice-pattern–selective TCA temporal factors were activated before the response and exhibited decreased activity after the response (Fig. 5*A*). Conversely, >50% of the outcome-selective TCA temporal factors showed an increase in ensemble activity following the response (Fig. 5*B*). On average, these functionally distinct TCA components displayed opposite trends in temporal factors before and after the response (Fig. 5*C*). We quantified these temporal differences between choice-pattern–selective and outcome-selective TCA components by comparing the peak time of the temporal factor. The peak time of the temporal factor of choice-pattern–selective TCA components (mean ± SD: left choice trials, −0.473 ± 1.78 s, right choice trials, −0.608 ± 1.51 s) was significantly earlier than that of outcome-selective TCA components (left choice trials, 0.696 ± 1.61 s, right choice trials, 0.525 ± 1.51 s; Mann–Whitney *U* test, left choice trials: *p *= 0.0189, right choice trials: *p *= 0.00401; Fig. 5*D*).

This finding suggests that the M2-DS ensemble plays a temporally distinct role in adaptive choice behavior by detecting outcomes after the choice response and flexibly making action decisions (such as repeating or switching choice) based on the outcome information.

### Comparison of TCA components based on M2- and DS-alone ensembles with TCA components based on M2-DS ensemble

If the features of ensemble activity of M2 and DS neurons were heterogeneous, or spike activity was not well coordinated between M2 and DS, the application of TCA to the concatenated ensemble activity of M2 and DS neurons (the M2-DS ensemble) could potentially attenuate task-related signals compared with TCA on ensemble activity in each region separately (Runyan et al., 2017). To explore this possibility, we investigated whether the choice-pattern–selective and outcome-selective TCA components derived from the M2-DS ensemble provided worse task-related signals than those obtained from ensembles in M2 or DS alone. TCA was separately applied to the M2-alone and DS-alone ensembles over the same data used for the M2-DS ensemble analysis. Choice-pattern–selective and outcome-selective TCA components were observed for both M2-alone and DS-alone ensembles. To control the number of neurons between M2-DS combined ensemble and M2-alone/DS-alone ensemble, we randomly resampled the equivalent number of neurons in the M2-DS ensemble relative to the number of neurons in M2-alone and DS-alone ensemble, respectively (Materials and Methods).

**Figure 6.**
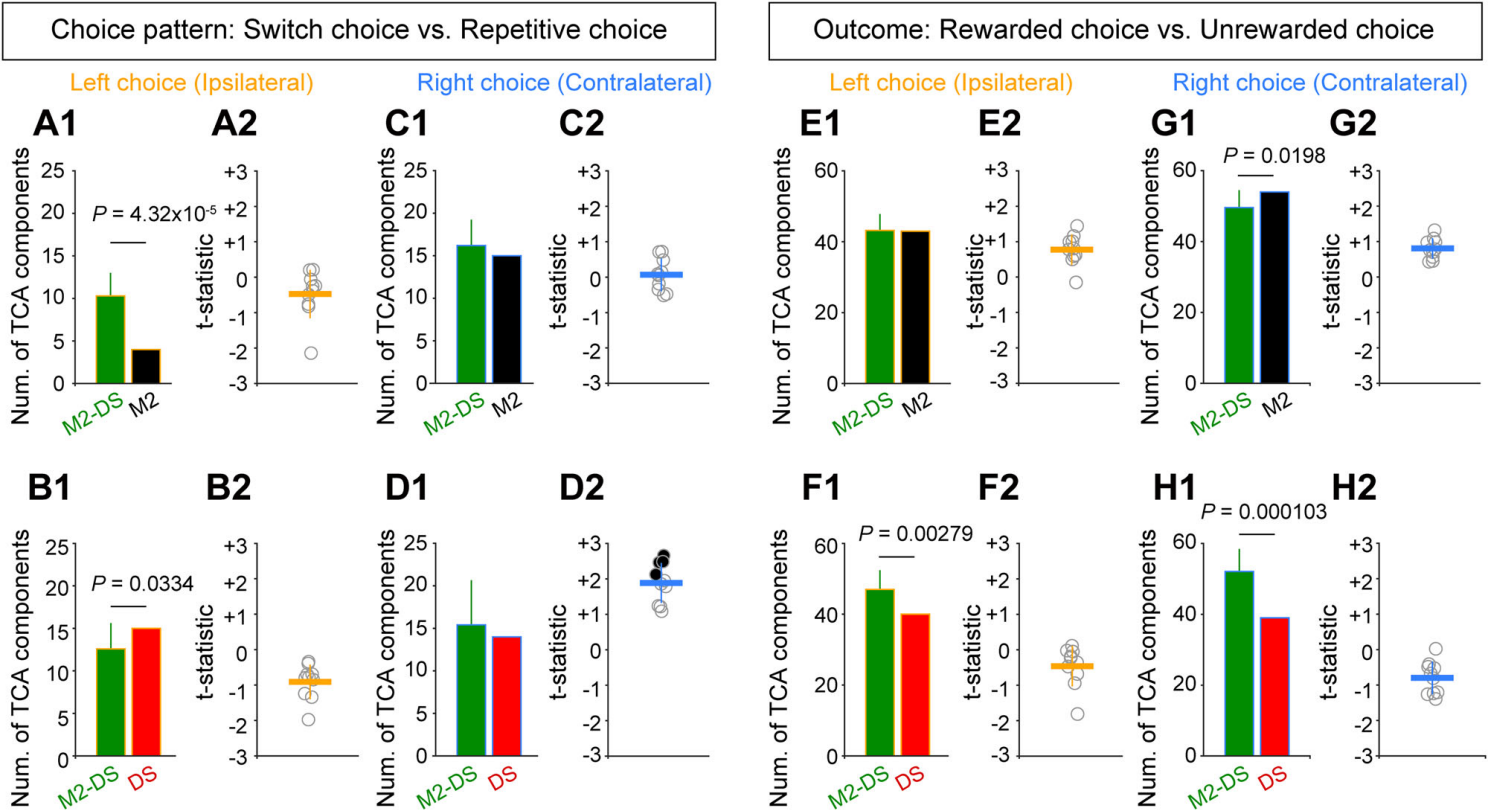
Comparison of TCA component based on M2-DS ensembles with those based on M2- or DS-alone ensemble. ***A–D***, Comparison of the total number of choice-selective TCA components (***A1***) between M2-DS ensembles (green) and M2-alone ensemble (black) and (***B1***) between M2-DS ensembles and DS-alone ensemble (red) in left choice block. These comparisons in right choice block (***C1***) between M2-DS ensembles and M2-alone ensemble and (***D1***) between M2-DS ensemble and DS-alone ensembles. The total number of TCA components in the M2-DS ensemble is presented by the mean and SD. *p* values were assessed by *t* test. ***A2***, ***B2***, ***C2***, ***D2***, The *t* statistic provided by two-sample *t* test of mean absolute *Z*-scores of trial factors at switch-selective TCA components between M2-DS ensemble and M2-/DS-alone ensembles. Open and filled symbols indicate statistical nonsignificance and significance, respectively (*t* test; *p *< 0.05). Horizontal and vertical solid lines represent mean and SD, respectively. ***E–H***, Comparison of the total number of outcome-selective TCA components (***E1***, ***G1***) between M2-DS ensembles and M2-alone ensemble and (***F1***, ***H1***) between M2-DS ensemble and DS-alone ensemble. ***E2***, ***F2***, ***G2***, ***H2***, The *t* statistic provided by two-sample *t* test of mean absolute *Z*-scores of trial factors at outcome-selective TCA components between M2-DS ensemble and M2-/DS-alone ensembles.

Regarding choice-pattern–selective TCA components, in left (ipsilateral) choice trials, the total number of detected TCA components was significantly fewer in the M2-alone ensemble (*n* = 4; *t* test: *p *= 4.32 × 10^−5^; Fig. 6*A1*), whereas that of the DS-alone ensemble (*n* = 15) was more than that of the M2-DS ensemble (*t* test: *p *= 0.0334; Fig. 6*B1*). In right (contralateral) choice trials, the number of choice-pattern–selective TCA components were comparable with that of the M2 (*n* = 15) and DS (*n* = 14) ensembles alone (*t* test: *p *= 0.244 for M2, *p *= 0.421 for DS; Fig. 6*C1*,*D1*). To compare the selectivity strength in the choice-pattern–selective TCA components between M2-DS ensemble and M2-/DS-alone ensembles, we compared the absolute *Z*-scores of the trial factors in switch choice trials between the M2-DS and M2-alone ensembles and between the M2-DS and DS-alone ensembles by two-sample *t* test to obtain *t* statistic. These values exhibited no significant difference by comparison with M2-alone ensemble (Fig. 6*A2*,*C2*). However, we found that the *Z*-scores of the trial factor were larger in M2-DS ensemble than those in DS-alone ensemble in right choice blocks although the statistical significance was detected in 4 out of 10 cases (Fig. 6*D2*, black symbols), but not in the left choice blocks (Fig. 6*B2*).

Regarding outcome-selective TCA components, the total number of TCA components based on the M2-DS ensemble were comparable with that based on the M2-alone ensemble (*n* = 43) in the left choice block (*t* test: *p *= 0.938; Fig. 6*E1*), whereas they were fewer than that based on the M2-alone ensemble (*n* = 55) in right choice block (*t* test: *p *= 0.0198; Fig. 6*G1*). Regarding the comparison with DS-alone ensembles, the number of TCA components in M2-DS ensemble were more than that in DS-alone ensemble (left/right choice: *n* = 40/39) in both blocks (*t* test: *p *= 0.00279 for left choice block; Fig. 6*F1*; *p *= 0.000103 for right choice block; Fig. 6*H1*). The selectivity strengths were not different between M2-DS ensemble and M2-/DS-alone ensembles in left and right choice blocks (Fig. 6*E2*,*G2*,*F2*,*H2*).

This finding indicates that TCA using the combined M2 and DS ensembles did not attenuate task-related signals in most cases; instead, it may not only reflect the contribution of either the M2 or DS ensemble but also the cooperative contribution of M2 and DS ensemble activity.

## Discussion

In this study, we demonstrated distinct representations of decomposed M2-DS ensemble activity for various task-relevant behaviors at the trial level in rats performing an outcome-based choice task using the TCA approach. TCA is an unsupervised method and, in contrast to linear discriminant analysis and linear regression, it automatically identifies choice-position, choice-pattern, and outcome-specific patterns. Choice-position–selective TCA components (the choice-position–specific spatiotemporal neural dynamics) revealed dynamic changes in selectivity over trials, which were correlated with changes in behavioral variables, such as RTs, and/or the number of licks after the response. The TCA revealed neural dynamics showing selectivity for choice patterns (repeat and switch choices), even when the choice position and outcome were identical. Choice-pattern–selective and outcome-selective neural dynamics revealed functionally distinct within-trial activity. Choice-pattern–selective within-trial activity increased activity earlier (before the choice response), whereas outcome-selective within-trial activity increased activity later (after the choice response). TCA application on M2-DS ensemble activity tended to yield more task-related neural dynamics than TCA application on M2 or DS alone.

The rodent M2 integrates sensory and outcome information to make decisions regarding motor planning (Barthas and Kwan, 2017). The activity of M2 neurons represents laterality in the body (Erlich et al., 2011; Sul et al., 2011), forelimb (Soma et al., 2017), and tongue movements (Siniscalchi et al., 2016; Handa et al., 2017, 2021; Kurikawa et al., 2018). Additionally, the M2 is implicated in outcome evaluation for action (Sul et al., 2011; Gremel and Costa, 2013; Handa et al., 2021) and in the neuronal encoding of outcomes, such as reward and nonreward events (Sul et al., 2011; Handa et al., 2021). The rodent DS serves a significant gateway of the basal ganglia, receiving major synaptic inputs from the cerebral cortex (Kincaid et al., 1998; Cheatwood et al., 2003; Reep et al., 2003; Wall et al., 2013; Hintiryan et al., 2016). Neuronal activity in DS similarly shows the laterality of the choice response (Kim et al., 2009; Handa et al., 2021) and the value of the outcome (Kim et al., 2009; Nonomura et al., 2018). Therefore, similar behavior-related signals are observed in the two regions. To gain a deeper understanding of the mechanisms underlying cross-region transmission between M2 and DS in outcome-based decision-making, a previous study used Fisher's linear discriminant (FLD) for dimensionality reduction of ensemble activity. The dynamic characteristics of the ensemble activity in each region were detected, and these characteristics were compared between M2 and DS. Neural trajectories in the M2 and DS revealed temporally similar task-related signals, such as choice position and outcome. Precise spike synchrony between M2 and DS neurons emerges more frequently when task performance is superior (Handa et al., 2021). Consistent with this result, another study demonstrated that cortical representation is topographically reflected in the striatal subregion (Peters et al., 2021). Our findings demonstrate that the M2-DS ensemble activity can be deconstructed into distinct functions—specifically, choice position, choice pattern, and outcome—without compromising the representation of decision-related signals in each region. This evidence suggests that the ensemble activity of interconnected regions (the M2-DS ensemble) effectively processes similar decision-related signals in a cooperative manner for further processing through downstream structures in the basal ganglia for motor selection.

Although the previous study successfully identified temporally parallel processing of information related to choice position and outcome between the M2 and DS using FLD, this approach encountered challenges in identifying choice-pattern–selective activity at the population activity level (Handa et al., 2021). Similar to conventional principal component analysis, FLD utilizes trial-averaged data to compute a vector of hyperplanes, maximizing the degree of discrimination between two conditions (left and right choices; Bishop, 2006). The assumption of trial averaging is that trial-by-trial variability is a task-irrelevant noise. Conversely, TCA considers trial-by-trial variability to extract features of population activity at a single-trial level in an unsupervised manner (Williams et al., 2018). Our current results not only demonstrated choice-position and outcome-selective M2-DS ensemble activity but also revealed choice-pattern–selective activity based on trial-by-trial analysis. These choice-pattern–selective neural dynamics were observed even in neural dynamics based on M2 or DS ensemble activity alone, suggesting that the choice pattern is commonly processed in both M2 and DS. In this case, dimensionality reduction in an unsupervised manner enables the uncovering of the latent features of the neural ensemble. Choice-pattern–selective neural dynamics of M2-DS ensemble activity were found more frequently in contralateral choice trials than in ipsilateral choice trials. In the contralateral choice trials, the selectivity strength of choice-pattern–selective TCA neural dynamics was larger in M2-DS ensembles than that in DS-alone ensemble. This result may reflect the cooperative contribution of M2-DS ensembles to the lateralized function. This intriguing component of the M2-DS ensemble activity may reflect cognitive features for action selection based on outcome rather than motor commands as the incoming choice position is identical, but the difference lies in selecting the spout position by deciding whether to repeat the previous choice or switch from the preceding choice in the outcome-based choice task. In support of this interpretation, previous studies have indicated that M2 is involved in cognitive switching between sensory-guided and automated choice rules (Siniscalchi et al., 2016), as well as flexible visual categorization (Wang et al., 2020). The DS is additionally implicated in the processing of action selection based on outcome probability or value (Nonomura et al., 2018; Cox and Witten, 2019). Our findings are consistent with these neuronal functions in both the M2 and DS, extending to M2-DS ensembles.

Choice-position–selective TCA neural dynamics revealed trial-to-trial fluctuations in choice-position selectivity, which correlated with variable behavioral parameters. The correlation with the number of licks may indicate the significance of the M2-DS ensemble activity in relation to changes in motivation to participate in the task, given the decrease in the number of licks observed in the later period of the session. Our finding suggests that the DS could be more of a contributor in the M2-DS circuit activity for the choice-position–selective TCA neural dynamics, whereas the M2 and DS contribute equally to the choice-pattern–selective and outcome-selective TCA dynamics. This newly observed result is also attributed to the trial-based TCA approach although we could not clarify the internal correlation between M2 and DS within M2-DS ensemble activity. The analysis of simultaneously recorded population activity is useful to interpret neural function related to behavioral or cognitive variables at the single-trial level (Kiani et al., 2014). Recent study demonstrates the relationship between trial-wise variability in choices and variability in value signals, which are related to decision-making, decoded from neuronal population activity in the nonhuman primate orbitofrontal cortex (McGinty and Lupkin, 2023). For the rodents, simultaneous recording of spiking activity of population of neurons from multiple brain regions could be applicable thanks to the recent technical advance in high-density electrode probes (Steinmetz et al., 2021). The read-out of neuronal functions across different brain regions by using such large-scale spike activity is important to interpret the functions of cross-regional neural circuits by means of the approach of dimensionality reduction together with cross-regional correlation analysis at the single trial level (Veuthey et al., 2020; Gokcen et al., 2022; Kondapavulur et al., 2022) in future studies.

A recent study demonstrated that synchronous spike activations across regions, including the medial prefrontal cortex and the downstream dorsomedial and ventral striatum, emerge with behavioral correlations contingent on task demands during the T-maze task. Different firing assemblies were observed at various times within the task (Oberto et al., 2022). In our study, the within-trial activity revealed distinct characteristics, such as choice-pattern–selective neural dynamics and outcome-selective neural dynamics, which varied temporally. This suggests that the M2-DS ensemble serves different functions at different time points. Choice-pattern–selective neural dynamics may be modulated before the choice response, influencing the decision to repeat or switch, whereas the outcome-selective component could be activated after the response to monitor the outcome. In essence, the M2-DS ensemble contributes to temporally distinct roles in adaptive choice behavior by detecting outcomes after choice response and flexibly making decisions of action (repeating or switching choices) based on the outcome information. Growing evidence indicates that frontal cortex-striatal ensemble coactivation emerges at specific times and plays specific roles in ongoing goal-directed behavior.

## Synthesis

Reviewing Editor: Mark Laubach, American University Decisions are customarily a result of the Reviewing Editor and the peer reviewers coming together and discussing their recommendations until a consensus is reached. When revisions are invited, a fact-based synthesis statement explaining their decision and outlining what is needed to prepare a revision will be listed below. The following reviewer(s) agreed to reveal their identity: Karunesh Ganguly.

Thanks for sending your paper to eNeuro! It was reviewed by two experts. Their reviews are given below in full. Please revise the paper to address all concerns that were raised. One of the reviewers in our forum made the comment below. It highlights the importance of explaining how you analyzed data from M2 and the striatum. Please be sure to address the comment below in your revised manuscript.

"...treating the neurons from the two regions as a single ensemble basically removes the inter-area dynamics, which may be the most interesting aspect of multi-region simultaneous recording experiments."

Also, your paper was submitted without a visual abstract. Please consider providing a graphic image that summarizes what you believe is the main new finding from your study. For example, you could use parts of Figure 1 (panel D) and Figure 2 (panel A). You could also use a plot of the results from the TCA that shows interactions between the two brain regions that are related to decisions.

Reviewer #1

This manuscript analyzes previously collected data with recordings from M2 and striatum during a task that requires flexibly switching between two ports. The authors then use TCA, a relatively newly developed analytical approach, to analyze the recorded data. TCA is unsupervised so it requires an interpretation of the low dimensional activity patterns into temporal/trial factors. Overall, this is a potentially nice descriptive analysis of the activity patterns. There are several points that need to be addressed.

1) The neuron factor shown seem to have different weights of DS and M2. For example, in 2B, there are large weighting in DS. This implies DS is more of a contributor. It would help to understand the different weighting in a systematic manner.

2) The activity from cortex and the striatum are treated as a single ensemble. Further, the bin size used was 200 ms. What is the justification for such a bin size? Conduction delays are likely a few ms and integration times may be < 20-50 ms. Is there evidence of more precise correlated activity across structures? This may also be due to the fact that relatively few neurons are sampled unlike with neuropixel.

3) Line 365. How overtrained were these animals? Is this trend found in all sessions per day?

4) The claim of a link between TCA and motivation states is not very clear from the figure and line 380-389 make a strong claim. Creating a new figure with more clear links would help; currently it seems more anecdotal.

5) Figure 2G2 - is the trend in lick rate a significant change? Perhaps a regression analysis can be done.

6) Figure 6 is a good control. I don't believe it controlled for the number of neurons. One way is to randomly sample the equivalent number of neurons in the combined ensemble relative to the number of neurons in M2 and DS. Does Fig 6 still hold up if done this way?

7) The activity from cortex and the striatum are treated as a single ensemble. In other words, the low dimensional patterns from each area are not separately considered in the optimization (e.g. CCA or dLAG will do this). This raises a fundamental question about how to view two connected areas. For example, one interpretation of the striatum is that it is an associative structure that can exist in more or less integrative mode. Is there evidence for coupling/uncoupling during the flexible switching?

Reviewer #2

In the present study, neural populations from cortico-striatal circuits (M2-Dorsal striatum) were recorded while rats executed an outcome-based action task. A left or right spigot was programmed to deliver reward in blocks of trials, such that reward delivery triggered a choice repeat, whereas a reward omission triggered a choice switch. Neural data was tensor coded (trials X neurons X trials) and Tensor Component Analysis was used to extract the magnitude of the population response across these dimensions. Trial-related components exhibited different magnitude in trials depending on the choice. Furthermore, the magnitude of this component predicted if the next choice would be a repeated choice or if there would be a switch, but this was lateralized and was seen only for switch toward the side contralateral to the recording site. These components also represented the outcome of the trial.

In addition, the authors show that neurons from M2 and Dorsal Striatum (DS) contribute to the representations of choice and outcome more or less evenly, whereas M2 neurons have a decreased contribution when the animals switch their choices to the side ipsilateral to the recording site (left).

It is an interesting and well executed study and provides appropriate support for the claims made.

My only major concern is related to the writing of the manuscript. I believe it could be heavily edited to a more concise form. There is a lot of redundant information (presented both in the Methods section and in the Results section). The sub-section "Statistics" within the methods section is redundant, too, because the tests mentioned have been already indicated in the previous sections.

Another aspect where the manuscript falls short is in the discussion of previous work showing population codes at the single trial level. I will not mention specific papers to avoid sounding prescriptive, but it should be easy to find relevant work in Prefrontal cortex, amygdala, and orbitofrontal cortex. Expanding the discussion on this aspect will further put the current work in context.

Other than that, I have only minor comments:

1) Lines 81-83: I understand that the reference was omitted here for double-blinding purposes, but please indicate the reference to the data source study in the final version of the manuscript.

2) Line 94: "Weekly, 10 ml of water was provided". Please confirm that this number is accurate, 10ml per week sounds like too little water.

3) Lines 131-133: "If the number of unrewarded choices increased within a block, block reversal occurred after many more trials". This is not clear. In the results section it is mentioned that animals had to accumulate >10 rewards (line 315) for the block to switch, but exactly how many? Was there a range?

4) Lines 184-192: This data would be better in a table; it is very difficult to follow in paragraph format. Also, what exactly is "reward acquisition probability"? Would it be equivalent to "fraction of correct choices"?

5) Lines 355-360: an example of the redundancies mentioned above, this was mentioned verbatim in the methods section.

6) Lines 408-410: "The lateralized difference between repetitive and switch choices suggests that choice-pattern selective neural dynamics did not account for the differences in the previous choices or outcomes between the choice patterns". It is unclear what this means.

7) I do not see any mention of figure 5D in the text. Did I miss it somewhere?

## Author Response

1 We thank all reviewers so much for all suggestions and their insightful comments which have helped us to improve the manuscript. Our point-to-point replies to reviewers' comments were written below. We highlighted all insertions and deletions indicated in the revised manuscript.

Response to Reviewer #1 We are grateful to the Reviewer #1 for his/her useful suggestions and comments to our original manuscript. We addressed his/her concerns and replied to the comments.

Synthesis Statement for Author (Required):

Thanks for sending your paper to eNeuro! It was reviewed by two experts. Their reviews are given below in full. Please revise the paper to address all concerns that were raised. One of the reviewers in our forum made the comment below. It highlights the importance of explaining how you analyzed data from M2 and the striatum. Please be sure to address the comment below in your revised manuscript. "...treating the neurons from the two regions as a single ensemble basically removes the inter-area dynamics, which may be the most interesting aspect of multi-region simultaneous recording experiments." Reply: We acknowledge this limitation, whereas we suggest the regional contribution to the decomposed neural dynamics and/or the cooperative contribution to the functional ensemble activity due to the additional analyses performed following the reviewer's suggestions. Our reply to the Reviewer's comment#7, which is related to this comment, includes the reply to this comment. Please refer to the reply below.

Reviewer #1 This manuscript analyzes previously collected data with recordings from M2 and striatum during a task that requires flexibly switching between two ports. The authors then use TCA, a relatively newly developed analytical approach, to analyze the recorded data. TCA is unsupervised so it requires an interpretation of the low dimensional activity patterns into temporal/trial factors. Overall, this is a potentially nice descriptive analysis of the activity patterns. There are several points that need to be addressed.

1) The neuron factor shown seem to have different weights of DS and M2. For example, 2 in 2B, there are large weighting in DS. This implies DS is more of a contributor. It would help to understand the different weighting in a systematic manner.

Reply: According to the reviewer's suggestion, to quantify the contribution of DS and M2 to the TCA component based on M2-DS combined activity, we computed the regional contribution index using neuron factor. The values of each neuron factor were separated into values for the M2 and DS cells and the root mean square (RMS) was computed for each region. The regional contribution index C was computed as follows.

$$C=(S_{\mathrm{DS}}-S_{M2})\;/(S_{\mathrm{DS}}+S_{M2}),$$

where SDS and SM2 indicate RMS of neuron factor for DS cells, and RMS of neuron factor for M2 cells.

To understand of regional contributions to the functional TCA components, we utilized this approach for choice-position selective TCA components (revised Figure 2I1 and 2I2), choice-pattern selective TCA components (revised Figure 3H), and outcome selective TCA components (revised Figure 4G) in a systematic manner. The regional contribution indices for choice-positive TCA components broadly ranged (revised Fig.

2I1 and 2I2, respectively). On average, the contribution index was significantly shifted to positive values, suggesting the DS were more of a contributor than the M2 (t-test:

TCA components correlated to RTs: P = 2.81x10-6 , TCA components correlated to the number of licks: P = 1.03x10-5 ). The regional contribution for choice-pattern selective TCA components indices broadly ranged (revised Fig. 3H). On average, the contribution index was not different from 0 (t-test: P = 0.135 for left choice block, P = 0.0911 for right choice block), suggesting the M2 and DS equally contributed to the choice-pattern selective TCA components. Regarding outcome-selective TCA components, the result was similar as that in choice-pattern selective TCA components.

The regional contribution indices broadly ranged in both left and right choice blocks (revised Fig. 4G). Mean contribution index was not different from 0 (t-test: P = 0.138 for left choice block, P = 0.0700 for right choice block), suggesting the M2 and DS equally contributed to the outcome selective TCA components.

We wrote the method for this new analysis in Materials and Methods (page 15, lines 339-345 of the revised manuscript), this result in Results (page 19, lines 433-439; page 21, lines 488-491; page 22, lines 511-514 of the revised manuscript), and their interpretation in the Discussion (page 29, lines 681-683 of the revised manuscript).

3 2) The activity from cortex and the striatum are treated as a single ensemble. Further, the bin size used was 200 ms. What is the justification for such a bin size? Conduction delays are likely a few ms and integration times may be < 20-50 ms. Is there evidence of more precise correlated activity across structures? This may also be due to the fact that relatively few neurons are sampled unlike with neuropixel.

Reply: This is because the analysis with 200 ms bin size more clarified the profile of TCA components. We analyzed the data at higher time resolution using 50-ms bin than the current version because we supposed that the transmission (or conduction delay) of neural signals between the M2 and DS takes a few milliseconds and integration times may be several tens of milliseconds order as the reviewer mentioned. As a result, the trend of characteristics in TCA components using 50-ms bin seemed to be more or less similar as the trends of results from the analysis using 200-ms bin, but we found some difficulties to profile the results. For example, the trial factor in TCA components of ensemble data with 50-ms bin exhibited the switch/repetitive choice pattern, but the difference between switch and repetitive choices was not clear due to increase of "noise trials", which was not correlated to this category (switch vs. repetitive choices).

Otherwise, this may be because these noise trials potentially reflected some other features. It was too complex (or noisy) to quantify the feature (or the pattern of trial factor) of TCA components with 50-ms bin resolution than the current TCA components with 200-ms bin resolution. This may be due to the small number of neurons in the neuronal ensemble in our dataset unlike neuronal ensemble recorded with Neuropixels, which is a high-density electrode probe including more than 5000 recording sites (Steinmetz et al., 2021), as the reviewer pointed out or due to the low spike rate of DS neurons. To profile the TCA components (especially trial factor) in our data, we concluded that 200-ms bin size was reasonable to analyze our data.

We mentioned this point in Materials and Methods as follows. "To obtain substantial firing activity in population of neurons, we chose the 200-ms bin for the sliding window because the signal-to-noise ratio in characteristics of TCA components using 200-ms bin was better than that analyzed using 50-ms bin for the sample size of our data." (page 10, lines 209-212 of the revised manuscript) Reference NA Steinmetz, C Aydin, A Lebedeva et al. Neuropixels2.0: A miniaturized high-density probe for stable, long-term brain recordings. Science, 372, eabf4588 (2021).

4 3) Line 365. How overtrained were these animals? Is this trend found in all sessions per day? Reply: We trained all rats over 21 training sessions (i.e., fixed number of training sessions across rats). If the performance of rats reached an achievement level (the reward acquisition probability of 75%) within the series of training sessions (e.g., 7 for R982-r1 as shown in the new Table 1), we defined the remaining sessions as overtraining sessions. Otherwise, there was no overtraining day (e.g., 0 for R997-r1 as shown in the new Table 1). After the training sessions, the rats were subject to recording experiments regardless of the learning achievement. As a result, there were various numbers of overtraining sessions.

Yes, we observed the trend in the choice-position selective TCA components in all sessions. Upon receiving the reviewer's comment, we examined the relationship between the property of choice-position selective TCA component and the number of overtraining sessions, but we did not find any relationship.

We added this new negative result in Result. (pages 18-19, lines 428-432 of the revised manuscript), the description about the definition for overtraining in Materials and Methods (page 7, lines 138-142 of the revised manuscript), and added the number of overtraining sessions in a new Table 1.

4) The claim of a link between TCA and motivation states is not very clear from the figure and line 380-389 make a strong claim. Creating a new figure with more clear links would help; currently it seems more anecdotal.

Reply: According to the reviewer's suggestion, we examined additional task parameter inter-trial intervals (ITIs), which were intervals between consecutive trials where animals were engaged in lick response post Go cue, as a motivational parameter. This is because we found that, if the rat did not respond to Go cue within the response window, the same trial was repeated until the rat made choice, which extended the duration of the ITI.

We found that the ITIs got longer at the late trials in the sessions (slope, mean 5 ±SD = 0.00659 ±0.00806, t-test: P = 0.00912). This increasing tendency of ITIs at the late trials indicates the reduction of motivation to engage in task performance. Thus, the ITIs could be a kind of behavioral variable to quantify how constantly animals were motivated to engage in choice action to obtain a reward. Then, we examined the relationship between the it is and the number of licks and found a significant correlation between the number of licks and ITIs and created new figures (revised Figure 2J and 2K). The number of licks were negatively correlated with the ITIs (Pearson's correlation coefficient: mean ±SD = -0.521 ±0.20751, t-test: P = 3.62x10-7 ) (revised Fig. 2J and 2K), but RTs were not (mean ±SD = 0.0307 ±0.262, t-test: P = 0.676) (revied Fig. 2K).

Therefore, the behavioral results suggest that this reduction in the number of licks over the trials may reflect changes in the motivational state of the rats to engage in task performance.

We added this description in Results (page 19, lines 440-452 of the revised manuscript) and the method in Materials and Methods (page 12, lines 260-266 of the revised manuscript).

5) Figure 2G2 - is the trend in lick rate a significant change? Perhaps a regression analysis can be done.

Reply: Yes, the decreasing trend in lick count was significant. Following the reviewer's suggestion, we checked the statistical significance using a regression model with t-test.

The slope of linear regression model, which was fitted to the trial series of the number of licks for each session, was negative (slope, mean ±SD = -0.00192 ±0.00101) and significantly different from 0 (t-test, P = 8.20x10-6 ).

We added this description in Results (page 18, lines 420-422 of the revised manuscript).

6) Figure 6 is a good control. I don't believe it controlled for the number of neurons.

One way is to randomly sample the equivalent number of neurons in the combined ensemble relative to the number of neurons in M2 and DS. Does Fig 6 still hold up if done this way? 6 Reply: According to the reviewer's suggestion, we controlled the number of neurons between M2-DS combined ensemble and M2-alone/DS-alone ensemble. We randomly resampled the equivalent number of neurons in the M2-DS ensemble relative to the number of neurons in M2-alone and DS-alone ensemble, respectively.

Regarding choice-pattern selective TCA components, in left (ipsilateral) choice trials, the total number of detected TCA components was significantly fewer in the M2-alone ensemble (n=4) (t-test: P = 4.32x10-5, revised Fig. 6A1), whereas that of the DS-alone ensemble (n=15) was more than that of the M2-DS ensemble (t-test: P = 0.0334, revised Fig. 6B1). In right (contralateral) choice trials, the number of choice-pattern-selective TCA components were comparable to that of the M2 (n=15) and DS (n=14) ensembles alone (t-test: P = 0.244 for M2, P = 0.421 for DS, revised Fig.

6C1 and 6D1). To compare the selectivity strength in the choice-pattern selective TCA components between M2-DS ensemble and M2- /DS-alone ensembles, we compared the absolute Z-scores of the trial factors in switch choice trials between the M2-DS and M2-alone ensembles and between the M2-DS and DS alone ensembles by two-sample t-test to obtain t-statistic. These values exhibited no significant difference by comparison with M2-alone ensemble (revised Fig. 6A2 and 6C2). However, we found that the Z-scores of the trial factor was larger in M2-DS ensemble than those in DS-alone ensemble in right choice blocks although the statistical significance was detected in 4 out of 10 cases (revised Fig. 6D2, black symbols), but not in the left choice blocks (revised Fig. 6B2).

Regarding outcome-selective TCA components, the total number of detected TCA components based on the M2-DS ensemble were comparable to that based on the M2-alone (n=43) in the left choice block (t-test: P = 0. 938, revised Fig. 6E1), whereas they were fewer than that based on the M2-alone (n=55) in right choice block (t-test: P = 0.0198, revised Fig. 6G1). Regarding the comparison with DS-alone ensembles, the number of TCA components in M2-DS ensemble were more than that in DS-alone ensemble (left/right choice: n=40/39) in both blocks (t-test: P = 0. 00279 for left choice block, revised Fig. 6F1; P = 0. 000103 for right choice block, revised Fig. 6H1). The selectivity strengths were not different between M2-DS ensemble and M2-/DS-alone ensembles in left and right choice blocks (revised Fig. 6E2, G2, F2, and H2).

This finding indicates that TCA using the combined M2 and DS ensembles did not attenuate task-related signals in most cases; instead, it may not only reflect the 7 contribution of either the M2 or DS ensemble but also the cooperative contribution of M2 and DS ensemble activity.

We added this method in Materials and Methods (page 14, lines 313-321 of revised manuscript) and rewrote the results in Results (pages 23-25, lines 550-594 of revised manuscript).

7) The activity from cortex and the striatum are treated as a single ensemble. In other words, the low dimensional patterns from each area are not separately considered in the optimization (e.g. CCA or dLAG will do this). This raises a fundamental question about how to view two connected areas. For example, one interpretation of the striatum is that it is an associative structure that can exist in more or less integrative mode. Is there evidence for coupling/uncoupling during the flexible switching? Reply: We treated the neuronal activities of M2 and DS as a single ensemble in this study because we aimed to view the activity of cortico-striatal circuit. This is because our previous work using the same spike data as the current study found the fine spike synchrony between M2 cell and DS cells during task performance. The proportion of M2-DS cell-pairs showing the spike synchrony was positively correlated to the task performance. Therefore, we consider that some functional coupling occurred between M2 cell and DS related to task performance although we did not figure out if the spike correlation was related to switch/repetitive choices.

To understand regional contribution to the TCA components, we analyzed the regional contribution index using neuron factor to functional TCA component according to the review's comment. This new result suggests that the DS could be a contributor in the M2-DS circuit activity on choice-position selective neural dynamics (revised Figure 2I1 and 2I2). This result suggests that the DS is an associative structure to integrate inputs from the M2 and potentially other areas. Additionally, the new analysis for the comparison of selectivity strength between the M2-DS ensemble and DS-alone ensemble indicates the cooperative contribution by M2-DS ensemble in some cases (revised Figure 6D2).

In sum, by using the combined M2-DS ensemble data, our results suggests that cross-regional population activity of connected-regions exhibited not only the properties of decomposed neural dynamics at single-trial level as well as the enhancement of 8 weighting of either of regions contributing to those functional neural dynamics or a cooperative contribution of two regions to the neural dynamics. The cooperative contribution of two regions to neural dynamics my be supported by the synchronous spike correlation between M2 and DS cells, although we could not clarify the internal correlation between M2 and DS within M2-DS ensemble activity unlike CCA and dLAG.

We discussed this point in Discussion (page 28, lines 655-660; page 29, lines 681-685 of the revised manuscript).

9 Responses to Reviewer #2 We are grateful to the Reviewer #2 for helpful suggestions to improve our paper.

Reviewer #2 In the present study, neural populations from cortico-striatal circuits (M2-Dorsal striatum) were recorded while rats executed an outcome-based action task. A left or right spigot was programmed to deliver reward in blocks of trials, such that reward delivery triggered a choice repeat, whereas a reward omission triggered a choice switch.

Neural data was tensor coded (trials X neurons X trials) and Tensor Component Analysis was used to extract the magnitude of the population response across these dimensions. Trial-related components exhibited different magnitude in trials depending on the choice. Furthermore, the magnitude of this component predicted if the next choice would be a repeated choice or if there would be a switch, but this was lateralized and was seen only for switch toward the side contralateral to the recording site. These components also represented the outcome of the trial.

In addition, the authors show that neurons from M2 and Dorsal Striatum (DS) contribute to the representations of choice and outcome more or less evenly, whereas M2 neurons have a decreased contribution when the animals switch their choices to the side ipsilateral to the recording site (left).

It is an interesting and well executed study and provides appropriate support for the claims made.

My only major concern is related to the writing of the manuscript. I believe it could be heavily edited to a more concise form. There is a lot of redundant information (presented both in the Methods section and in the Results section). The sub-section "Statistics" within the methods section is redundant, too, because the tests mentioned have been already indicated in the previous sections.

Reply: According to the reviewer's suggestion, we deleted this section because all description about statistical tests was written in previous section. (pages 14-15, lines 321-336 of the revised manuscript) 10 Another aspect where the manuscript falls short is in the discussion of previous work showing population codes at the single trial level. I will not mention specific papers to avoid sounding prescriptive, but it should be easy to find relevant work in Prefrontal cortex, amygdala, and orbitofrontal cortex. Expanding the discussion on this aspect will further put the current work in context Reply: According to the reviewer's suggestion, we discussed more about previous and recent works using population activity at the single trial level in Discussion as follows.

The analysis of simultaneously recorded population activity is useful to interpret neural function related to behavioral or cognitive variables at the single-trial level (Kiani et al., 2014). Recent study demonstrates the relationship between trial-wise variability in choices and variability in value signals, which are related to decision-making, decoded from neuronal population activity in the non-human primate orbitofrontal cortex (OFC) (McGinty and Lupkin, 2023). For the rodents, simultaneous recording of spiking activity of population of neurons from multiple brain regions could be applicable thanks to the recent technical advance for high-density electrode probes (Steinmetz et al., 2021). The read-out of neuronal functions across different brain regions by using such large-scale spike activity is important to interpret the functions of cross-regional neural circuits by means of the approach of dimensionality reduction like our study together with cross-regional correlation analysis at the single trial level (Veuthey et al., 2020; Gokcen et al., 2022; Kondapavulur et al., 2022) in future studies. (page 29, lines 685-697 of the revised manuscript) We added the following papers in References of the revised manuscript.

References Gokcen E, Jasper AI, Semedo JD, Zandvakili A, Kohn A, Machens CK, Yu BM (2022) Disentangling the flow of signals between populations of neurons. Nat Comput Sci 2:512-525.

Kiani R, Cueva CJ, Reppas JB, Newsome WT (2014) Dynamics of Neural Population Responses in Prefrontal Cortex Indicate Changes of Mind on Single Trials. Curr Biol 24:1542-1547.

Kondapavulur S, Lemke SM, Darevsky D, Guo L, Khanna P, Ganguly K (2022) Transition from predictable to variable motor cortex and striatal ensemble patterning during behavioral exploration. Nat Commun 13:2450.

McGinty VB, Lupkin SM (2023) Behavioral read-out from population value signals in 11 primate orbitofrontal cortex. Nat Neurosci 26:2203-2212.

Steinmetz NA et al. (2021) Neuropixels 2.0: A miniaturized high-density probe for stable, long-term brain recordings. Science 372:eabf4588.

Veuthey TL, Derosier K, Kondapavulur S, Ganguly K (2020) Single-trial cross-area neural population dynamics during long-term skill learning. Nat Commun 11:4057.

Other than that, I have only minor comments:

1) Lines 81-83: I understand that the reference was omitted here for double-blinding purposes, but please indicate the reference to the data source study in the final version of the manuscript.

Reply: For the purpose of double-blinding review, we had removed the reference.

Although we still need to remove the reference in the revised manuscript for double-blinding re-review process, we included the reference in a Clean Copy of the revised manuscript, which could be a final version if accepted.

2) Line 94: "Weekly, 10 ml of water was provided". Please confirm that this number is accurate, 10ml per week sounds like too little water.

Reply: This was not an accurate number. We rephrased the sentence to express the accurate number with information about when and how much the rats obtained water in Materials and Methods as follows. (page 5, lines 96-98 of the revised manuscript) "Rats obtained about 10 ml of water at the task chamber when they engaged in the task performance, whereas they were supplied 10 ml of water at the cage when the behavioral experiment was not performed." 3) Lines 131-133: "If the number of unrewarded choices increased within a block, block reversal occurred after many more trials". This is not clear. In the results section it is mentioned that animals had to accumulate >10 rewards (line 315) for the block to switch, but exactly how many? Was there a range? 12 Reply: Ten accumulated rewarded trials was a fixed criterion to reverse the reward block. If the rats frequently selected the incorrect spout, the block reversal occurred though more than 11-12 trials because the block reversal occurred when the rat achieved the criterion (10 rewarded choices).

We rewrote this explanation in Materials and Methods (page 7, lines 135-137 of the revised manuscript).

Therefore, there was no range of criteria. We made a typo in Results of the previous version of our manuscript (">10" was wrong) and led this confusion. We corrected this typo in the text as follows. "The action-outcome association was systematically reversed without sensory feedback after accumulating 10 rewarded trials in each block." (page 15, lines 351-352 of revised manuscript):

The averaged trial number per block was 12.5 ± 0.650 (mean ± SD). We added this new data in the text (page 16, lines 356-357 of the revised manuscript).

4) Lines 184-192: This data would be better in a table; it is very difficult to follow in paragraph format. Also, what exactly is "reward acquisition probability"? Would it be equivalent to "fraction of correct choices"? Reply: Yes, the reward acquisition probability is equivalent to the fraction of correct choices. Following the reviewer's suggestion, we summarized the information about our data set in new Table 1. Thus, we rewrote the description about the data set in Materials and Methods as follows. "Data set for this study is summarized in Table 1, where the experimental ID, number of overtraining sessions, reward acquisition probability (the fraction of correct choices) in the recording session, and number of M2 and DS units are listed." (page 9, lines 201-204 of the revised manuscript) 5) Lines 355-360: an example of the redundancies mentioned above, this was mentioned verbatim in the methods section.

13 Reply: We edited this redundant sentence to avoid repetitive description in Results as follows.

Previous version: "TCA decomposes the M2-DS ensemble activity into a third order tensor $X_{ntk}=\sum_{r=1}^{R}\;w_{n}^{r}b_{t}^{r}a_{k}^{r}$ by the summation of one-rank component (Fig. 2A).

Each component comprises three vectors: $w_{n}^{r}$ is the n-th element of a 'neuron factor' vector, representing a prototypical firing rate pattern across neurons (Fig. 2B); $b_{t}^{r}$ is the t-th element of a 'temporal factor' vector, indicating a temporal basis function across time (Fig. 2C); and $a_{k}^{r}$ is the k-th element of a 'trial factor' vector, serving as a trial-specific bias for spatiotemporal activity in a trial (Fig. 2D)." New version: "TCA decomposes the M2-DS ensemble activity into a third order tensor (Fig. 2A). Each component comprises three vectors, including a 'neuron factor' vector (Fig. 2B), a 'temporal factor' vector (Fig. 2C), and a 'trial factor' vector (Fig. 2D)." (page 17, lines 393-399 of the revised manuscript) 6) Lines 408-410: "The lateralized difference between repetitive and switch choices suggests that choice-pattern selective neural dynamics did not account for the differences in the previous choices or outcomes between the choice patterns". It is unclear what this means.

Reply: If the difference in Z-scores between switch and repetitive choices reflected difference in previous choice positions (or previous outcomes), the difference in Z-score should be observed in both left and right choice conditions. Therefore, this lateralized difference between repetitive and switch choices could not be attributed to the differences in the previous choices or outcomes between the choice patterns, suggesting that this difference may reflect rather a lateralized cognitive function for switch or repetitive choices.

We rewrote these sentences (pages 20-21, lines 475-481 of revised manuscript).

7) I do not see any mention of figure 5D in the text. Did I miss it somewhere? Reply: No, the reviewer did not miss it. Because Figure 5D was not mentioned in the previous version of our manuscript, we mentioned the Figure 5D in the revised version 14 of manuscript as below. "The peak time of the temporal factor of choice-pattern selective TCA components (mean ±SD: left choice trials, -0.473 ±1.78 s, right choice trials, -0.608 ±1.51 s) was significantly earlier than that of outcome-selective TCA components (left choice trials, 0.696 ±1.61 s, right choice trials, 0.525 ±1.51 s) (Mann-Whitney U-test, left choice trials: P = 0.0189, right choice trials: P = 0.00401) (Fig. 5D)." (page 23, lines 529-533 of the revised manuscript)
